# Control, Elimination, and Eradication of River Blindness: Scenarios, Timelines, and Ivermectin Treatment Needs in Africa

**DOI:** 10.1371/journal.pntd.0003664

**Published:** 2015-04-10

**Authors:** Young Eun Kim, Jan H. F. Remme, Peter Steinmann, Wilma A. Stolk, Jean-Baptiste Roungou, Fabrizio Tediosi

**Affiliations:** 1 Swiss Tropical and Public Health Institute, Basel, Switzerland; 2 University of Basel, Basel, Switzerland; 3 Consultant, Ornex, France; 4 Erasmus MC, University Medical Center Rotterdam, Rotterdam, The Netherlands; 5 African Programme for Onchocerciasis Control, Ouagadougou, Burkina Faso; Centers for Disease Control and Prevention, UNITED STATES

## Abstract

River blindness (onchocerciasis) causes severe itching, skin lesions, and vision impairment including blindness. More than 99% of all current cases are found in sub-Saharan Africa. Fortunately, vector control and community-directed treatment with ivermectin have significantly reduced morbidity. Studies in Mali and Senegal proved the feasibility of elimination with ivermectin administration. The treatment goal is shifting from control to elimination in endemic African regions. Given limited resources, national and global policymakers need a rigorous analysis comparing investment options. For this, we developed scenarios for alternative treatment goals and compared treatment timelines and drug needs between the scenarios. Control, elimination, and eradication scenarios were developed with reference to current standard practices, large-scale studies, and historical data. For each scenario, the timeline when treatment is expected to stop at country level was predicted using a dynamical transmission model, and ivermectin treatment needs were predicted based on population in endemic areas, treatment coverage data, and the frequency of community-directed treatment. The control scenario requires community-directed treatment with ivermectin beyond 2045 with around 2.63 billion treatments over 2013–2045; the elimination scenario, until 2028 in areas where feasible, but beyond 2045 in countries with operational challenges, around 1.15 billion treatments; and the eradication scenario, lasting until 2040, around 1.30 billion treatments. The eradication scenario is the most favorable in terms of the timeline of the intervention phase and treatment needs. For its realization, strong health systems and political will are required to overcome epidemiological and political challenges.

## Author Summary

River blindness (onchocerciasis) is transmitted by blackflies and causes severe itching, skin lesions, and vision impairment including blindness. More than 99% of all current cases are found in sub-Saharan Africa where the disease has historically hindered socioeconomic development in endemic areas. The treatment goal is shifting from control to elimination in Africa as morbidity has significantly decreased through vector control and community-directed treatment with ivermectin. Studies in Mali and Senegal proved that elimination is feasible with ivermectin administration. Given limited resources, national and global policymakers need a rigorous analysis of investment options from epidemiological, economic, and societal aspects. For this, we developed control, elimination, and eradication scenarios and compared treatment timelines and drug needs over the next 30 years. We found that the elimination and eradication scenarios would require a shorter treatment phase and a smaller amount of ivermectin than the control scenario, mainly because community-directed treatment with ivermectin could be ended earlier thanks to regular active surveillance.

## Introduction

Elimination of neglected tropical diseases (NTDs) has recently emerged on the global health agenda and gained prominence with the release of the global plan to combat NTDs by the World Health Organization (WHO) [1]. In 2012,WHO issued a roadmap towards the elimination of 17 NTDs [2], and stakeholders from the public and private sectors pledged to contribute to the control, elimination, and eradication of ten NTDs through the London Declaration on NTDs [3]. The second WHO report on NTDs further elaborated the roadmap [4], and the London Declaration follow-up report showed the substantial progress that had already been achieved through the stakeholder partnership approach [5].

One of the NTDs targeted for elimination is onchocerciasis (river blindness). This is a parasitic disease caused by filariae that are transmitted by blackflies. Severe itching, skin lesions, and vision impairment including blindness are its most notable symptoms. Onchocerciasis is endemic in parts of Africa, Latin America, and Yemen, but over 99% of all current cases are found in sub-Saharan Africa [6] where onchocerciasis has historically been a serious public health problem and hindered socioeconomic development in endemic areas [7]. However, many infections are asymptomatic, and vector control and community-directed treatment with ivermectin have significantly reduced morbidity. Specifically, the Onchocerciasis Control Program (OCP), which was implemented in West Africa from1975 to 2002, and the African Programme for Onchocerciasis Control (APOC), which has supported onchocerciasis control activities in sub-Saharan countries since 1995 and continued the OCP’s activities where needed, have decreased the burden of disease to such an extent that it is no longer a public health problem in most endemic areas [8]. In Latin America, the Onchocerciasis Elimination Program for the Americas (OEPA) implemented since 1993 has brought the disease close to elimination. Colombia and Ecuador announced the elimination of onchocerciasis after WHO verification in 2013 and 2014, respectively [9,10]. Treatment has also been stopped in seven foci in Guatemala and Mexico where it has been replaced by surveillance to detect possible recrudescence [11]. Regional elimination in Latin America is expected to be achievable by 2020 if the regular treatment of a sufficient proportion of the nomadic Yanomami in the border area between Brazil and Venezuela can be achieved [12]. In Yemen, onchocerciasis is endemic in a limited number of communities. Elimination in the near future is considered technically feasible, and a national action plan aiming at elimination by 2015 was developed in 2010 [13]. Currently, political instability and security concerns that limit access to endemic areas hamper its implementation [4].

Studies in Mali and Senegal have proved the feasibility of onchocerciasis elimination through ivermectin treatment in some hyper-endemic foci in West Africa [14,15]. This has provided additional momentum and arguments for a shift in the strategic goal from control to elimination also in Africa. The decision to invest in elimination and eradication efforts should be informed by broad assessments considering biological and technical feasibility, financial and economic costs, health and economic gains, capacity of and impacts on health systems, and societal and political willingness to cooperate [16]. An approach to such an assessment has been proposed in the form of eradication investment cases in 2010 [17]. Tediosi and colleagues have examined the approach with focus on three NTDs including onchocerciasis [18]. With reference to this approach, we have developed and compared alternative scenarios, namely, staying in a control mode versus moving toward elimination and subsequent eradication.

In the present paper, we describe the scenarios to achieve control, elimination, and eradication of onchocerciasis, predict the timeline of stopping treatment at country level, and estimate the number of required ivermectin treatments over the next 30 years with focus on Africa.

## Methods

### Development of scenarios

We developed *scenarios*, describing all required activities and resources that are expected to lead to the goals of control, elimination, and eradication, if effectively implemented and sustained as long as required, based on current standard practice, the results of large-scale studies, and available historical data. To clearly distinguish these alternative scenarios, we referred to the definitions of control, elimination, and eradication endorsed and recommended by the WHO Strategic and Technical Advisory Group for NTDs [19]. The ultimate goals of the scenarios were defined as follows:

1) control scenario: continuing community-directed treatment with ivermectin (CDTi) to keep the prevalence under a locally acceptable level; 2) elimination scenario: scaling up CDTi to all endemic areas where feasible aiming at the reduction of disease incidence to zero; and 3) eradication scenario: including strategies and tailored interventions to overcome operational challenges in endemic areas with feasibility concerns in addition to CDTi with the aim of reducing the global disease incidence to zero (Table 1).

**Table 1:** Proposed scenarios of control, elimination, and eradication of onchocerciasis.

	**Control**	**Elimination**	**Eradication**
Ultimate goal	Reduce disease prevalence to a locally acceptable level	Reduce the incidence of infection to zero in a defined geographical area	Reduce the worldwide incidence of infection to zero
**Target areas**			
Endemicity	Hyper, meso	Hyper, meso, hypo	Hyper, meso, hypo
Feasibility concerns for CDTi^1^	Partially targeted	Partially targeted	Targeted^2^
**Activities at project level**			
**Phase 1. Intervention**			
1. Community-directed treatment with ivermectin (CDTi)			
Frequency	Once a year	Once or twice^3^ a year	
Treatment coverage	65%+	65%+	
Start year of new projects^4^	2014–2015	2014–2015: hyper-/meso-endemic	2014–2015: hyper-/meso-endemic
		2016–2017: hypo-endemic	2016–2017: hypo-endemic, with no feasibility concerns for CDTi
			2020–2021: hypo-endemic, with feasibility concerns for CDTi
Duration	25 years; another 25 years in case of insufficient treatment coverage	Until the probability of local elimination is ≥ 99%^5^	
2. Surveillance			
Type	Epidemiological	1A) Epidemiological	
		1B) Epidemiological and entomological	
Frequency	Last year of MDA (25^th^, 50^th^ year)	1A) Every 4 years from 9^th^ year of MDA	
		1B) Last one year	
Site	10 villages	1A) 10 villages	
		1B) 20 villages (epidemiological surveys) and 4 catching sites (entomological)	
**Phase 2. Confirmation of elimination**			
Surveillance			
Type	NA	Epidemiological and entomological	
Frequency	NA	Epidemiological: last one year (3^rd^ year)	
	NA	Entomological: last two years (2^nd^ and 3^rd^ year)	
Site	NA	10 villages and 4 catching sites	
**Phase 3. Post-elimination**			
Surveillance			
Type	NA	Epidemiological and entomological	
Frequency	NA	Epidemiological: every 3 years	
	NA	Entomological: every 4 years	
Site	NA	5 villages and 2 catching sites	

^1^ Political insecurity and co-endemicity with *Loa loa*.

^2^ Hypo-endemic areas with feasibility concerns were included in the eradication scenario only.

^3^ Twice a year in new projects in Ethiopia and Uganda where the respective ministries of health announced six-monthly CDTi in new projects to bring them in line with ongoing projects [20,21]

^4^ Predicted considering APOC’s strategic plan to focus on the onchocerciasis elimination for the next decade 2016–2025 and the current epidemiological and political situation

^5^ A dynamical transmission model ONCHOSIM [22] was used.

From an operational perspective, the control and elimination scenarios are designed to target endemic areas where interventions appear feasible without major challenges, whereas the eradication scenario is an optimal situation. To make the eradication scenario feasible, intensive efforts to improve operational capacity and to increase political willingness would be required to overcome epidemiological and political challenges. We assume effective treatment would be implemented through tailored approaches in those areas, and regular surveillance would be maintained during and after the intervention phase until eradication has been verified.

Referring to the general principles for developing scenarios outlined by Tediosi and colleagues [18], the key components of scenarios were identified at project level. Scenarios were further revised by verifying the realism of assumptions in consultation with a technical advisory group consisting of policymakers, onchocerciasis epidemiologists, public health experts, health economists, and donors.

Key components for developing the scenarios are defined as follows and the developed scenarios are described in Table 1.

#### Projects

In all APOC areas, operational decisions regarding drug administration and monitoring are made at the level of projects whose geographic scope ranges from a single district to a whole country, and which is under the leadership of a project management team supported by the ministry of health, APOC, and NGOs [23]. For the purpose of modeling, all ongoing and potential new projects were identified and counted. First, a total of 112 projects were identified to be active in 16 APOC countries as of November 2013 based on the APOC treatment database (last update: 2012). Additional potential endemic areas in APOC countries that are not yet covered by systematic ivermectin treatment and hence not part of existing projects were identified using the Rapid Epidemiological Mapping of Onchocerciasis (REMO) map [24] and broken down into 43 potential new projects considering administrative boundaries, endemicity, *Loa loa* (African eyeworm) co-endemicity, and operational feasibility. For endemic countries in former OCP countries, a provisional database had been set up with the information on geographical location, pre-control endemicity, latest available treatment coverage, and population at project level. Treatment areas were divided into hypothetical project areas based on administrative boundaries, treatment history, and available impact evaluation data. A total of 17 such projects in 10 endemic countries in West Africa were identified as ongoing as of November 2013. Two possibly endemic regions in Côte d’Ivoire and Ghana had reportedly implemented neither CDTi nor vector control; thus they were included as new projects in the database.

#### Population

The population for ongoing projects (as of November 2013) was derived from the APOC treatment database and the provisional database for former OCP countries. The information came from the census conducted by community drug distributors for estimating drug needs. For potential new projects, the population was estimated by multiplying the surface area (km^2^) of the project with the average population density (per km^2^) across other projects with census data within the country. Population over the next 30 years was adjusted for population growth rates [25].

#### Pre-control endemicity

For APOC countries, endemicity was classified into four levels based on the highest pre-control nodule prevalence among adult males in the project area, namely, non-endemic with less than 5% nodule prevalence, hypo-endemic between 5% and 20%, meso-endemic between 20% and 40%, and hyper-endemic with 40% and above. The pre-control geographic distribution of nodule prevalence in each project area was obtained from a map generated using a kriging analysis of REMO survey results [26]. For former OCP countries, endemicity was classified based on the highest pre-control microfilariae prevalence among the population aged 5 years and older, namely, non-endemic with less than 10% microfilariae prevalence, hypo-endemic between 10% to 40%, meso-endemic between 40% and 60%, and hyper-endemic with 60% and above. The pre-control geographical distribution of microfilariae prevalence in each project area was obtained from a map generated using a kriging analysis of pre-control skin snip survey results. The corresponding ranges of microfilariae prevalence to nodule prevalence for the endemicity levels were estimated using a published relationship between microfilariae and nodule prevalences [27].

#### Community-directed treatment with ivermectin

CDTi was considered the primary treatment approach. Ivermectin is known as a safe drug to treat early onchocerciasis symptoms and prevent lasting symptoms from developing to blindness. WHO deemed that non-medical people could administer ivermectin after training [28–30], and APOC formally adopted CDTi in 1997 [31]. In this approach, community volunteers play a key role; they conduct a community census to determine the required amount of ivermectin, plan when and how to distribute ivermectin in their communities, administer the correct dose of ivermectin, manage adverse reactions, keep records, and report to health workers.

#### Target projects

Target projects were selected for each scenario based on the pre-control endemicity and considering the treatment goals. The control scenario included projects with meso- and hyper-endemicity considering the goal of keeping the prevalence under a locally acceptable level. The fact that surrounding hypo-endemic areas are not treated implies the possibility of recrudescence, yet was considered consistent with the aim of control as onchocerciasis is not a public health problem in hypo-endemic areas.

The elimination scenario extended the target projects to include those in hypo-endemic areas. The target projects in hypo-endemic areas, however, were confined to those where CDTi is expected to be operationally feasible at present or in the near future. To assess the feasibility, we referred to the criteria defined by the International Task Force on Disease Eradication and agreed in the Ernst Strüngmann Forum [32]. First, for biological and epidemiological feasibility, we took into account co-endemicity with *L*. *loa*, as severe adverse reactions against ivermectin can occur in *L*. *loa* patients with heavy infection, and the availability of alternative treatment approaches to mitigate the risk. For social and political feasibility, we took into account the current political situation and previous project performance.

The eradication scenario further extended the target areas to include all projects in hypo-endemic areas assuming locally tailored approaches would be successfully employed in the areas with epidemiological and political feasibility concerns.

#### Frequency of CDTi

Annual CDTi was assumed considering CDTi had been conducted annually in most projects in Africa [8]. Exceptions were new projects in Ethiopia [20] and Uganda [21] where the respective ministries of health announced six-monthly CDTi in new projects to bring them in line with ongoing projects.

#### Treatment coverage

Treatment coverage was defined as the proportion of the total population residing in a project area that was actually treated. APOC suggests that the treatment coverage needs to be higher than 65% for the program to achieve effective control of the disease [33]. The average treatment coverage over the last three years (2010–2012) was calculated based on the APOC treatment database and assumed to be stable at that level for the future duration of CDTi. If a project had, on average, not achieved the recommended coverage (65%), we assumed that future treatment coverage would be equal to the highest coverage that had been achieved over 2010–2012. In case historical data were lacking, the national average treatment coverage over projects with available data was used. If there were no projects with available historical data within a country, the regional average (over national averages available) for the APOC regions was used. In the database for former OCP countries, only the latest treatment coverage data were available and were used as the expected treatment coverage. The treatment coverage data in the former OCP countries were higher than 65%, and we used the national average for new projects.

#### Start year of CDTi

The historical start years of ongoing projects were obtained from the APOC treatment database. For projects yet to be started, a start year was predicted based on APOC’s strategic plan to focus on onchocerciasis elimination for the next decade 2016–2025 [34], the current epidemiology, and the current political situation. A start year in 2014–2015 was assumed for projects in areas without feasibility concerns. For other projects with feasibility concerns, two phases were distinguished depending on the current epidemiology. Projects in meso- and hyper-endemic areas without feasibility concerns were assumed to start CDTi in 2016–2017, because these areas were expected to be given priority considering the regional momentum toward onchocerciasis elimination. Projects in hypo-endemic areas with feasibility concerns were assumed to start in 2020–2021, as countries are not likely to postpone treatment by more than a decade if they actually aim at eradication. Within each of the three groups, the year when a project is expected to start CDTi was determined using a point system in which the earlier year is assigned if the project has a higher level of endemicity, a larger population size, and a higher expected treatment coverage, considering CDTi is expected to be more urgently needed if the disease is more prevalent and more people are exposed to the risk of infection, and also more feasible if the treatment compliance is expected to be higher compared to other projects.

#### Duration of CDTi

In the control scenario, the treatment goal is to control the disease as a public health problem by CDTi in all meso- and hyper-endemic areas where there is a high risk of disease. The required duration of CDTi was assumed to be 25 years considering that ONCHOSIM simulations predict that 25 years of annual ivermectin would achieve elimination in highly endemic areas (pre-control microfilariae per skin snip: 50 to 70 mf/s) and all areas with lower endemicity levels. Local elimination might occur within 25 years; however, the lack of regular surveillance to evaluate progress and verify elimination entails continued CDTi. This is the current practice in former OCP regions, that is, CDTi has been continued until now although the disease had been eliminated as a public health problem in most areas when OCP stopped in 2002. For 14% of the projects targeted in the control scenario, an additional 25 years of CDTi was assumed, referring to the results of APOC's most recent evaluations that showed unsatisfactory decline in infection levels in 14% of the evaluated projects (5 of 35) due to insufficient treatment coverage [35]. The projects requiring this additional CDTi effort were randomly selected regardless of the historical treatment coverage, because the APOC evaluation revealed that some projects that had reported high treatment coverage had actually failed to maintain the coverage above 65%.

In the elimination and eradication scenarios, the required duration of CDTi was predicted using ONCHOSIM which uses a stochastic model to simulate the life events of human individuals and inhabitant parasites and a deterministic model to simulate the fly dynamics and the development of parasites in the flies [22]. The model had been fitted to longitudinal data from Ghana [36,37], and the predicted trends of infection had been shown to be consistent with the actually observed trends in the study sites in Mali and Senegal [14,15,38]. The model estimated the years of CDTi required for achieving elimination with a probability of 99% by simulating the dynamics of transmission for different settings with regard to pre-control endemicity and treatment coverage.

#### End year when CDTi can be stopped

The year when CDTi can be stopped was estimated by adding the required CDTi duration to the start year. If the predicted end year was before 2014, it was delayed to 2015 or 2016, as stopping treatment is likely to be cautiously ordered at present despite epidemiological and entomological evidence indicating that the threshold for safely stopping CDTi had been reached. To date, little practical experience has been collected in this domain, and restarting CDTi is considered more challenging than maintaining CDTi a few years beyond the actually required time. The number of years for which stopping CDTi was delayed was determined using a point system in which the end year was more delayed if the project had a higher level of pre-control endemicity, a larger population size, and a lower treatment coverage than other projects. The rational was that more solid evidence would be needed to stop CDTi if the pre-control endemicity had been higher, more people were exposed to the risk of infection, and the treatment compliance had been lower than in other projects.

#### Surveillance

Two types of surveillance were assumed: epidemiological surveillance to track infection levels in the population and entomological surveillance to evaluate the infectivity rate of blackflies.

The control scenario included epidemiological surveillance only in the expected end year of CDTi to confirm that the infection level was low enough to stop CDTi. This reflects the practice under the control mode until recently in most endemic African regions, which do not have routine surveillance systems as the goal has been controlling the disease rather than eliminating it.

For the elimination and eradication scenarios, surveillance strategies with three phases were defined based on the conceptual and operational framework of onchocerciasis elimination with ivermectin treatment developed by APOC [39] and in consultation with the technical advisory group. For detailed activities, we referred to a protocol for epidemiological surveillance developed by APOC and a guide for post treatment surveillance produced by OEPA [40]. In phase 1, the intervention phase, epidemiological surveys are scheduled every 4 years, starting after 8 years of CDTi. The aim is to assess the impact of treatment and the prevalence decline towards elimination thresholds. In the expected final year of CDTi, entomological as well as epidemiological surveys are assumed in all endemic areas to confirm that elimination thresholds have been reached and CDTi can be safely stopped. Following the confirmation that the prevalence and the vector infectivity rate have reached the thresholds to stop CDTi, phase 2 starts. Its goal is to confirm local elimination and it lasts at least three years. In this phase, epidemiological surveys in the last year and entomological surveys in the last two years are planned to confirm that the infection prevalence and the vector infectivity rate continue to decrease toward zero and that no recrudescence has occurred. In phase 3, the post-elimination phase, surveillance consists of epidemiological surveys every 3 years and entomological surveys every 4 years, but less intensive than in phase 2 (e.g., a smaller number of survey sites). The objective is to detect possible recrudescence until eradication has been verified. If surveillance detects recrudescence after CDTi had been stopped, phase 1 restarts with a focus on the area where the recrudescence had happened and adjacent areas.

### Number of required ivermectin treatments

The number of required ivermectin treatments to achieve the goals of the control, elimination and eradication scenarios in endemic African regions was predicted by multiplying the estimated population living in endemic areas with the treatment coverage rate and the CDTi frequency per year for the required duration of treatment at project level. The capacity of drug manufacturers to supply the required number of ivermectin was assumed to be sufficient considering Merck’s commitment to donate ivermectin until elimination is achieved globally [41].

The time horizon for predicting the number of treatments was 2013 to 2045. The start year was set considering the most recent version of the APOC databases available for analysis was for 2012. The end year was chosen based on the prediction that the last project in the eradication scenario would stop CDTi in 2040, and that after stopping CDTi, at least three years would be required to confirm local elimination. In the control and elimination scenarios, the last projects were expected to continue CDTi beyond 2045.

In the S1 Table, the relevant data regarding the key components of the scenarios, which were used for estimating the timelines and the number of required ivermectin treatments, are presented at project level.

### Uncertainty analysis

Parameters used for the scenario analysis were subject to considerable uncertainty and the impact of the uncertainty was examined for the target population, the timeline when CDTi is expected to be stopped, and the number of required ivermectin treatments. The impact of a single parameter’s uncertainty was assessed with one-way deterministic sensitivity analysis (DSA). Considering the final estimates are driven by the joint effects of multiple parameters, multivariate probabilistic sensitivity analysis (PSA) was conducted with all the variables examined in the one-way DSA.

The included parameters were population growth rate, treatment coverage, treatment duration, CDTi start and end years, and the assumptions for selecting target projects. For DSA, the parameter uncertainty ranges were determined based on available data, expert opinion or both. For PSA, statistical distributions were chosen considering the characteristics of parameters, and fitted to available data. Simulations were run 1,000 times for each scenario.

#### Population growth rate

For DSA, the range of national population growth rates from the UN database was used [25]. For PSA, a normal distribution was fitted, assuming the range of population growth rates to be the 95% confidence interval.

#### Treatment coverage

The range of uncertainty about treatment coverage was assumed to be ±10% of the expected coverage in DSA. For PSA, a beta distribution was selected considering treatment coverage is between zero and one, and fitted to the historical data over 2010–2012. In these sensitivity analyses, samples were bounded from 60% to 84%, because control and elimination is not expected to be achievable with treatment coverage less than 60%, and the maximum achievable coverage is 84% considering around 16% of the population in endemic regions is not eligible for treatment because individuals are less than five years old, pregnant, or severely ill.

Average treatment coverage data and distribution parameters are presented in the S2 Table, and distribution graphs are shown in S1 Fig.

#### CDTi duration

For DSA and PSA, the CDTi duration was linked to the treatment coverage so that it changed automatically with the variation of treatment coverage. For the relationship between treatment coverage and CDTi duration, we used the results of ONCHOSIM [22] simulations fitted to the longitudinal data from Ghana [36,37]. For the control scenario, the CDTi duration changed only if the required duration predicted by the ONCHOSIM simulation was longer than 25 years; otherwise 25 years of CDTi was assumed as previously described.

#### Delay in starting and ending CDTi

Starting and ending CDTi could be delayed as political turmoil or operational difficulties arise or become exacerbated. Also, CDTi might be stopped later than expected considering APOC has strict criteria for ending CDTi (e.g., prevalence of mf < 1% in 90% of surveyed villages) [39]. For DSA, the uncertainty range of the delay in starting and ending CDTi was assumed to be from 0 to 5 years. For PSA, a gamma distribution that has around 90% of samples between 0 and 5 was selected (S2 Fig).

#### Selection of target projects

In the control scenario, 14% of the target projects were assumed to need another 25-year of CDTi due to insufficient treatment coverage as previously described. In DSA, the proportion of projects that were expected to have insufficient treatment coverage was varied between 0% and 14% under the control scenario, and in PSA a uniform distribution was used.

The elimination and eradication scenarios included 18 potential new projects in hypo-endemic areas (S1 Table). Onchocerciasis mapping based on REMO surveys has been largely completed in the APOC countries [24]. However, nodule palpation may give false positive results in non-endemic areas, while most REMO surveys were done more than 10 to 15 years ago before the start of CDTi. Recent parasitological surveys to determine the current infection status of hypo-endemic areas [42] have shown that, in many of these areas, onchocerciasis is no longer endemic. In order to take this into account, the number of new projects in hypo-endemic areas was varied between 20% and 100% of the total number of potential new projects: the lower bound was based on the finding that one of five potential project areas was confirmed to be hypo-endemic, and the upper bound based on the possibility that all potential hypo-endemic areas could be confirmed to be endemic. For PSA, a uniform distribution was used.

## Results

For each scenario, we predicted target areas in endemic African regions and population in those areas, the timeline when CDTi is expected to be stopped, and the number of required ivermectin treatments.

### Target areas and population

The control scenario targeted hyper-and meso-endemic areas in all endemic African countries. Under the elimination scenario, CDTi was extended to hypo-endemic areas where CDTi is feasible in addition to hyper-and meso-endemic areas. Countries that include projects with feasibility concerns have been identified to be the Central African Republic, the Democratic Republic of the Congo, and South Sudan due to political instability, and Gabon due to the high prevalence of *L*. *loa* in areas with a low prevalence of onchocerciasis. In these four countries, hypo-endemicity areas were therefore excluded from the elimination scenario. The eradication scenario targeted all hyper-, meso-, and hypo-endemic areas. The endemic countries in Africa were categorized into two control programs in which they participate or participated, APOC and OCP, respectively (Table 2).

**Table 2:** Endemic countries in Africa.

**Program**	**Endemic countries**
**APOC**	Angola, Burundi, Cameroon, the Central African Republic*, Chad, Congo, the Democratic Republic of the Congo*, Equatorial Guinea, Ethiopia, Gabon*, Liberia, Malawi, Mozambique**, Nigeria, South Sudan*, Sudan, Tanzania, Uganda (total 18 countries)
**Former OCP**	Benin, Burkina Faso, Côte d'Ivoire, Ghana, Guinea, Guinea Bissau, Mali, Senegal, Sierra Leone, Togo (total 10 countries)

* countries with epidemiological or political insecurity issues

** non-endemic with possible exception of small border areas with Malawi and Tanzania

**Fig 1:**
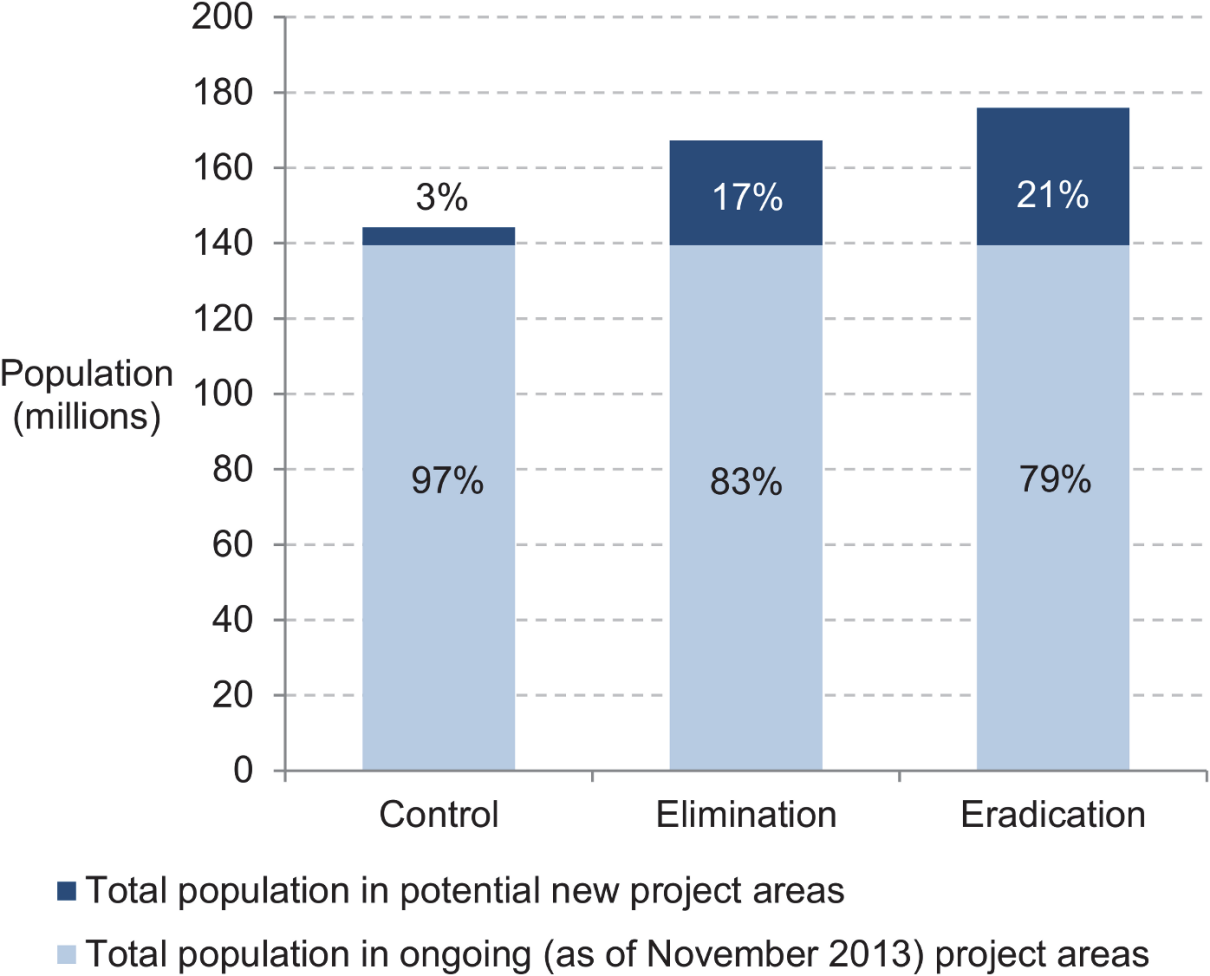
Total population living in ongoing and potential new project areas in endemic African countries (numbers, % of total population in endemic regions), 2014.

The control scenario included 27 countries, and potential new projects were predicted to cover around 3% of the total population in the entire target area, or 4.7 million of 144 million (Fig 1). The elimination scenario included the same 27 countries, and new projects were predicted to cover at most 17% of the population in the entire target area (167 million). Depending on the number of new projects in potential hypo-endemic areas, the population in new project areas ranged from 12.1 million to 27.8 million (7% and 17%). The eradication scenario included one more country, Gabon, and the total population in the entire target area was estimated at around 176 million of which 21% at maximum live in new project areas with a range of 12.1 million to 36.5 million people (7% to 21%) depending on the number of new projects in potential hypo-endemic areas.

### Expected year when CDTi can be stopped

**Fig 2:**
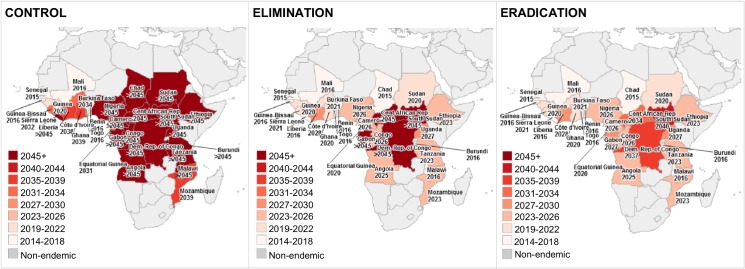
Years when CDTi is expected to be stopped in endemic African regions.

**Fig 3:**
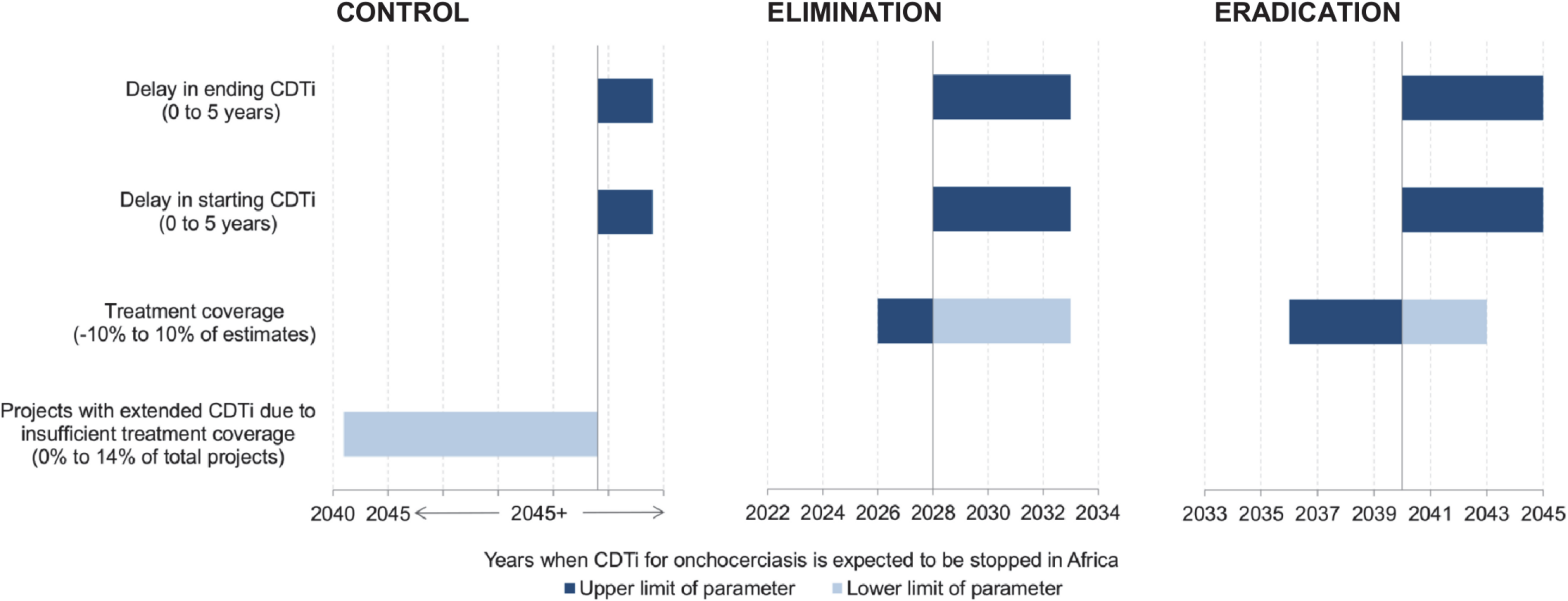
One-way deterministic sensitivity analysis for the years when CDTi is expected to be stopped in endemic African regions. CONTROL also applies to the countries with feasibility concerns in the elimination scenario. ELIMINATION excludes countries with feasibility concerns.

In the control scenario, most endemic countries outside West Africa were predicted to continue CDTi beyond 2045 (Fig 2). The most influential parameter determining the expected year of ending CDTi was the extension of treatment duration due to insufficient treatment coverage (Fig 3). For the elimination and eradication scenarios, the final year of CDTi represents the year of ending the intervention phase at country level assuming no recrudescence would occur. In the elimination scenario, all endemic countries except the four countries with feasibility concerns were expected to finish the intervention phase by 2028 at the latest and those four countries were expected to continue CDTi beyond 2045 (Fig 2). In the eradication scenario, all endemic countries were expected to reach the end of the intervention phase by 2040 assuming sufficient treatment would be delivered sustainably in the four countries with epidemiological and political concerns. For the elimination and eradication scenarios, one-way DSA (Fig 3) showed that any delay in starting and ending CDTi and low treatment coverage would result in the intervention phase to end later than expected; on the contrary, high treatment coverage would expedite the progress of the intervention phase and lead to an earlier end of the intervention phase.

### Number of required ivermectin treatments

**Fig 4:**
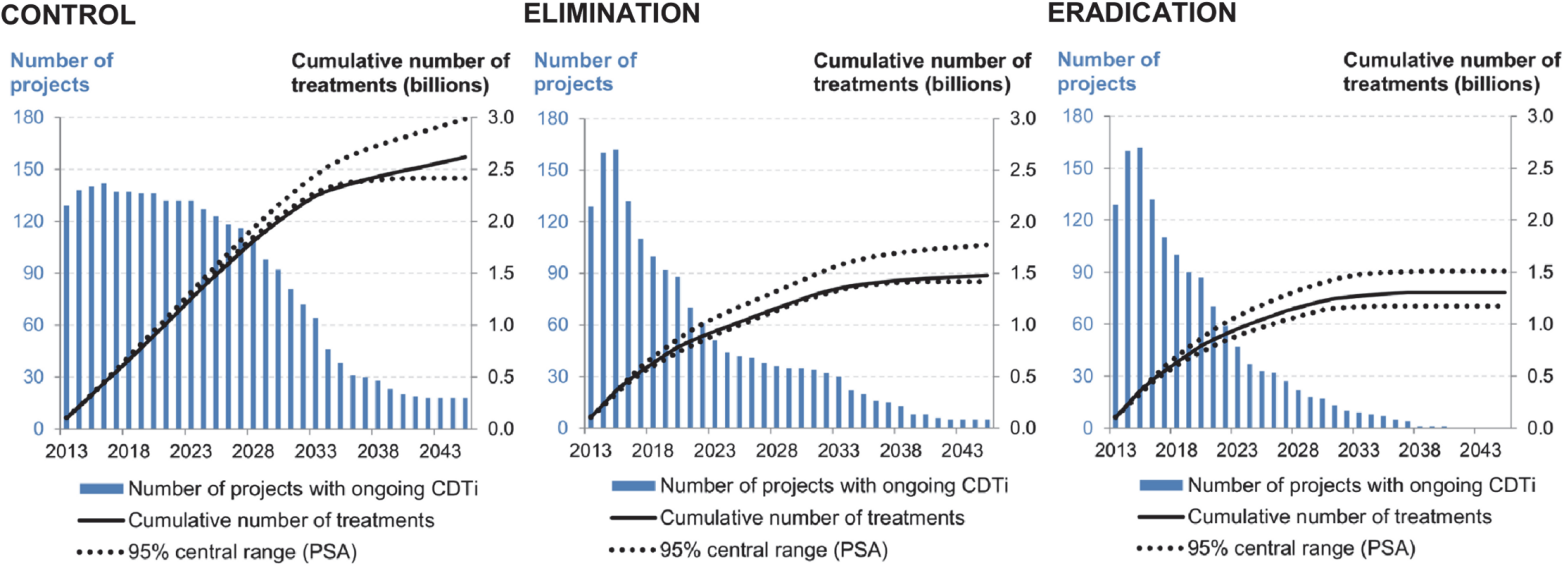
Cumulative number of ivermectin treatments and annual number of projects with ongoing CDTi in endemic African regions, 2013–2045.

**Fig 5:**
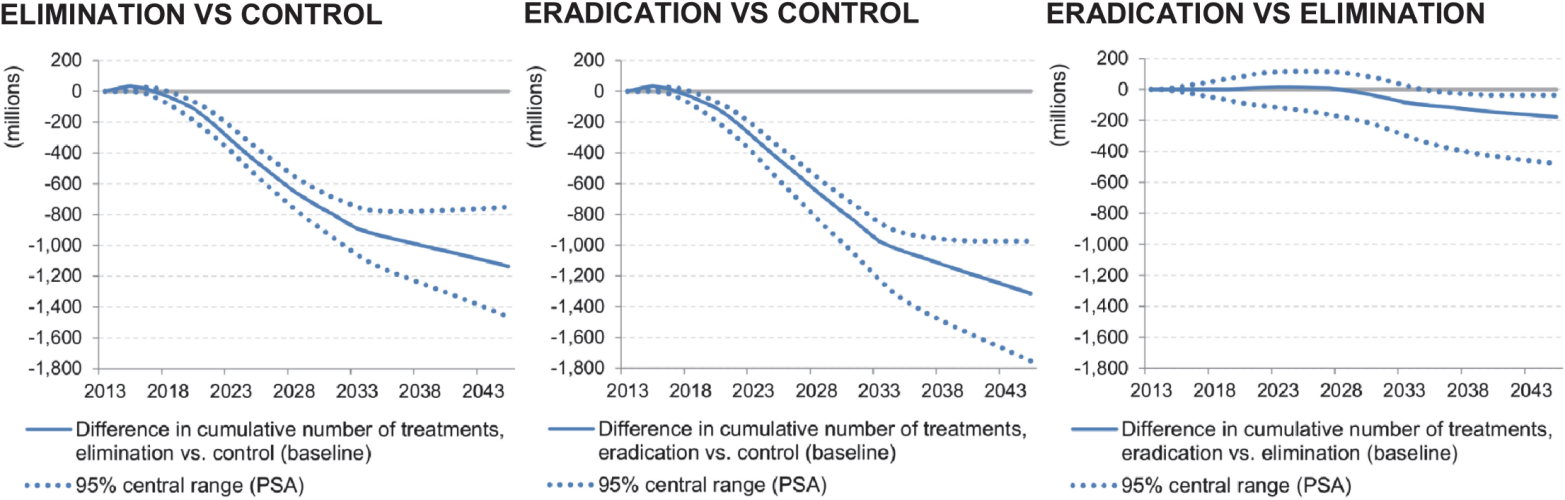
Difference in the cumulative number of ivermectin treatments between scenarios, 2013–2045.

**Fig 6:**
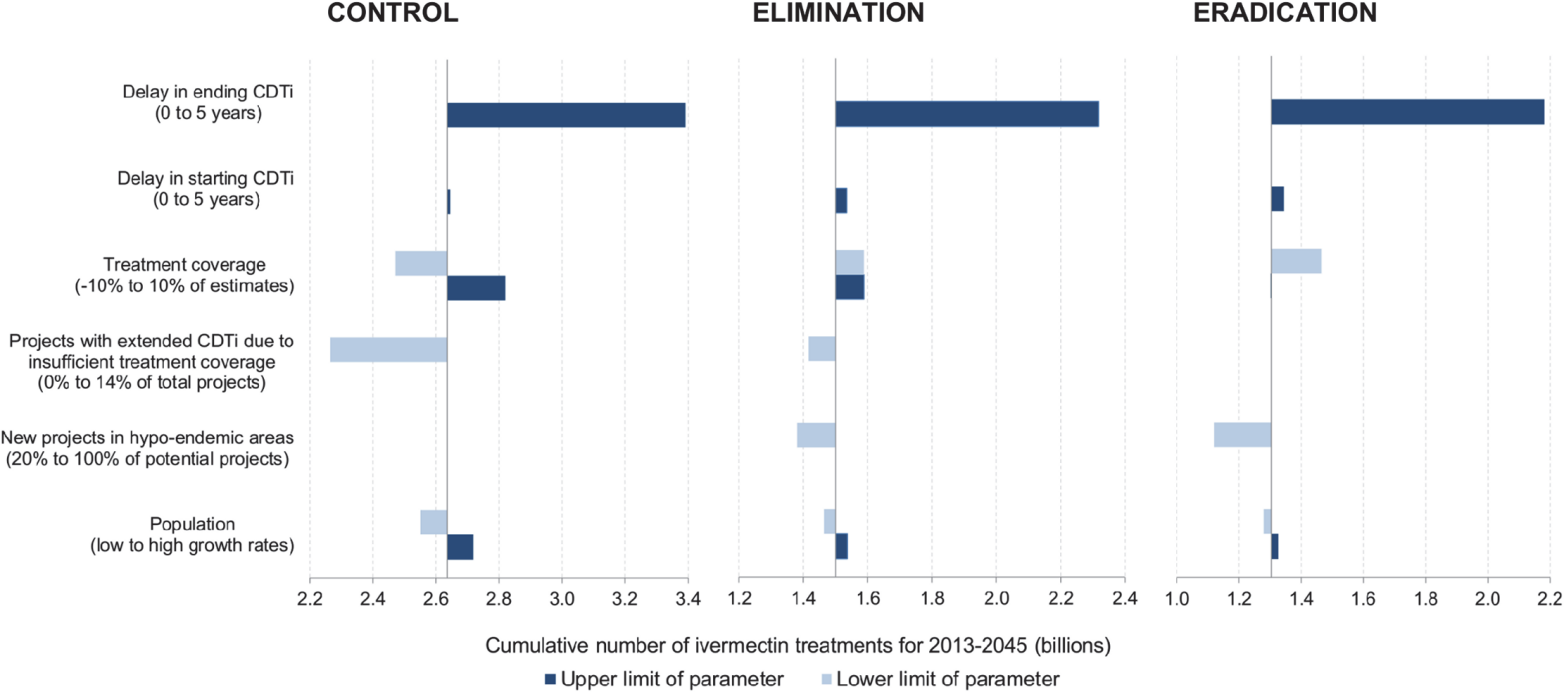
One-way deterministic sensitivity analysis for the cumulative number of ivermectin treatments over 2013–2045.

The need for ivermectin treatments was concentrated in the first half of the time horizon for the elimination and eradication scenarios, as 80% of all potential projects were stopped safely by 2031 and 2025, respectively. In the control scenario, it took until 2038 for the same proportion of the total projects to stop CDTi (Fig 4). The cumulative number of required ivermectin treatments over 2013–2045 was estimated at 2.63 billion (95% central range: 2.41 billion-2.99 billion) for the control scenario. Specifically, 1.48 billion (1.51bn-1.57bn) treatments were predicted to be required until 2025 and 1.15 billion (0.90bn-1.41bn) treatments over 2026–2045 (Table 3). According to the simulation of the elimination scenario, the required number of ivermectin treatments over the whole period was around 1.48 billion (1.42bn-1.79bn). Compared to the control scenario, the total number of required treatments in the elimination scenario was lower by 1.15 billion (44%): 0.45 billion (0.36bn-0.55bn) until 2025 and 0.69 billion (0.38bn-0.92bn) from 2026 to 2045 (Table 3, Fig 5). The eradication scenario required an even smaller number of ivermectin treatments for the whole period, 1.30 billion (1.18bn-1.51bn), which was 0.18 billion (0.03bn-0.49bn), or 12%, lower than that under the elimination scenario and 1.32 billion (0.97bn-1.75bn), or 50%, lower than that under the control scenario (Fig 5). In one-way DSA (Fig 6), the most influential parameter on the cumulative number of required ivermectin treatments was the delay in ending CDTi in all scenarios. For the control scenario, the second most influential parameter was the number of projects with extended CDTi duration due to insufficient treatment coverage. For the elimination and eradication scenarios, it was the number of potential new projects in hypo-endemic areas.

**Table 3:** Population in target areas and the cumulative number of required ivermectin treatments in endemic African regions.

	Control	Elimination	Eradication
**2013–2025**			
Population living in target areas, 2025	189,958,000	217,377,000	229,557,000
Cumulative number of required ivermectin treatments	1,480,765,000	1,027,466,000	1,041,229,000
**2026–2035**			
Population living in target areas, 2035	238,794,000	273,380,000	289,519,000
Cumulative number of required ivermectin treatments	859,636,000	367,629,000	249,291,000
**2036–2045**			
Population living in target areas, 2045	293,373,000	336,005,000	357,428,000
Cumulative number of required ivermectin treatments	287,319,000	86,630,000	12,681,000

## Discussion

The key changes for shifting from the control mode to elimination and subsequent eradication are the scale-up of CDTi to hypo-endemic areas and the implementation of regular epidemiological and entomological surveys along with ongoing surveillance. For successful implementation of these, overcoming the existing feasibility issues related to the co-endemicity with *L*. *loa*, the insecure political situation, and weak health systems will be critical. We found that, if this could be accomplished, regional elimination in Africa could be achieved as early as 2040, and consequently all endemic countries including Latin Americas and Yemen would be in the post-elimination phase until eradication has been verified.

We found that achieving elimination would reduce treatment needs by 43% compared to the control mode for the period 2013–2045. The driver of this remarkable difference is that CDTi could be stopped for the majority of projects based on regular surveillance, while it would have to continue for at least 25 years under the control scenario. The eradication scenario is predicted to require an even smaller number of ivermectin treatments than the elimination scenario, as hypo-endemic areas with feasibility concerns were assumed to have a shorter treatment period through effective treatment via tailored approaches as well as CDTi, whereas those areas would be under the control mode in the elimination scenario. This finding implies that saved ivermectin drugs could be used for other disease programs, for instance, mass drug administration (MDA) for lymphatic filariasis (LF).

The uncertainty about the target population in the elimination and eradication scenarios was mainly driven by uncertainty in the number of potential new projects in hypo-endemic areas, as some of those areas might not be actually endemic. Parasitological surveys are therefore needed to determine the current infection status of those areas. Setting up a new project requires operational planning, human resource mobilization, and startup costs. To move towards elimination without delay and to save human and financial resources, the rapid mapping of potential hypo-endemic areas should be a priority to confirm areas to set up new projects and to develop elimination strategies for those areas.

The main driver of the number of required ivermectin treatments was the delay in stopping CDTi. This finding implies that maintaining high treatment coverage to avoid the extension of treatment duration and continuous monitoring and evaluation to decide a proper time to stop CDTi would lead to faster elimination and prevent unnecessary efforts to deliver drugs.

We assumed no recrudescence in our analysis. However, if recrudescence occurs, the duration of CDTi would need to be extended, local elimination would be delayed, and the number of required treatments would increase. Recrudescence might occur because of human or vector migration, interrupted drug distribution due to political instability, and residual transmission from not-treated endemic areas due to incomplete or inconsistent geographic coverage.

We did not adjust for alternative treatment approaches for areas where *L*. *loa* is highly endemic but onchocerciasis is hypo-endemic. Suggested treatment approaches for these areas include anti-*Wolbachia* therapy with macrofilaricidal drugs, high doses of albendazole, and the test-and-treat strategy [43,44]. These approaches would expedite elimination and increase the demand for other drugs while reducing the need for ivermectin.

Our modeling did not incorporate the impact of changing the CDTi frequency on the treatment duration. It has been suggested to increase the frequency of CDTi to reduce the prevalence and transmission of onchocerciasis faster compared to the annual CDTi [35]. A recent study by Coffeng and colleagues shows that six-monthly ivermectin treatment could reduce the required treatment duration by 40% based on a dynamical transmission model [38]. In practice, increasing the CDTi frequency would require collaboration between policymakers, health workers, and community volunteers and new strategies on how to mobilize human and financial resources, given limited resources and competing health programs. Under the control scenario, annual CDTi could mean overtreatment for projects that had more than 15–20 years of treatment, for example, some areas in West Africa where ivermectin administration has been implemented since the 1990s. For these areas, less frequent CDTi could be an alternative for morbidity control, which would require a smaller number of ivermectin and less human and financial resources. However, less frequent CDTi might lead to a loss of local expertise, human resources, and community compliance over the time interval without CDTi and, consequently, to the decrease of treatment coverage below the required level, which could expose the areas to the risk of recrudescence.

We did not incorporate possible delays in ending CDTi due to co-endemicity with LF. In areas where LF is co-endemic with onchocerciasis, an assessment whether both diseases have reached the thresholds to stop treatment will be needed in order to stop CDTi. In practice, no delay is expected in most cases as MDA for LF, which relies on albendazole and ivermectin, usually requires fewer cycles to reach the point of transition to the post-treatment phase. However, LF mapping or anti-LF MDA have not started in about a third of the 35 endemic countries in Africa [45].

We did not take into account the possibility of drug resistance, as no confirmed cases of ivermectin resistance have been reported from endemic countries so far. However, if ivermectin resistance were to happen as suggested by Bourguinat and colleagues through studies on the effects of ivermectin on the genetics of *Onchocerca volvulus* [46], the entire efforts for onchocerciasis treatment could be endangered, as current strategies heavily rely on ivermectin.

The long time horizon of 2013–2045 poses challenges in predicting technological, political, and economic changes. New treatment and diagnostic tools could be game changers in achieving elimination. Ivermectin is a microfilaricidal drug which requires many years of treatment and has a risk of eliciting severe adverse reactions in *L*. *loa* patients. Macrofilaricidal drugs that are safe and effective for general population use, are easy to administer in communities, and have a shorter treatment period than ivermectin could substantially change treatment strategies and expedite elimination. Several macrofilaricidal drugs for human use have been or currently are under development, e.g., doxycycline [47], emodepside [48], moxidectin [49], and flubendazole [50]. The need for diagnostic techniques that are capable of detecting infections early, are easy to use in the field, and are affordable would greatly facilitate surveillance when early detection of new infections is paramount. The skin snip method, currently the most common diagnostic method, has low sensitivity for detecting very light infections, and can result in a delay in detecting recrudescence. Several diagnostic techniques, e.g., OV-16 (ELISA and Rapid Test) and the DEC patch test [51,52], that may prove more sensitive and practical, have been developed. Unexpected political unrest might hamper the elimination programs, as it interrupts interventions and weakens political support. Industrialization along with economic growth may have a significant impact. For instance, the construction of dams can flood existing breeding sites of blackflies or create new ones, and deforestation can greatly alter the composition or density of blackfly populations.

Political will across the whole spectrum of stakeholders from global and national policymakers to community members will be particularly critical during the “last mile” towards elimination and subsequent eradication [53]. Countries sharing borders spanning endemic areas would need to effectively collaborate to enable prompt responses to or prevent possible recrudescence. Regular meetings have been held between Guinea/Sierra Leone/Liberia, Togo/Benin, and Benin/Nigeria [54], and this proves such mechanism can work. Similar collaborative relationships would need to be fostered for other endemic countries. APOC has announced that it would transform to a new regional entity by 2016 that would support integrated country-driven programs to eliminate onchocerciasis, LF, and other preventive chemotherapy NTDs (soil-transmitted helminthiasis, schistosomiasis, trachoma) in Africa [55,56]. Successful launching of this new regional entity might provide a more collaborative environment for sustainable interventions and post-treatment surveillance for NTDs in the region. Continuous support from community members is essential for onchocerciasis elimination in Africa. National policymakers would need to keep empowering community drug distributors, as their role is critical for successful CDTi and will continue to be so until eradication has been achieved.

## Supporting Information

S1 Table Population, onchocerciasis endemicity, feasibility concern for community-directed treatment with ivermectin (CDTi), start year and frequency of CDTi, treatment coverage, and predicted end year of CDTi for ongoing (as of November 2013) and potential new projects. (PDF)

S2 Table Summary of treatment coverage and distribution parameters for probabilistic sensitivity analysis for endemic African countries. (PDF)

S1 Fig Beta distributions for treatment coverage for endemic African countries. (TIF)

S2 Fig Probability and cumulative density graphs for the gamma distribution applied to the delay in starting and ending CDTi. (TIF)

## References

[1] WHO (2007) Global plan to combat neglected tropical diseases, 2008–2015 WHO/CDS/NTD/2007.3. Geneva: WHO http://whqlibdoc.who.int/hq/2007/WHO_CDS_NTD_2007.3_eng.pdf Accessed on 25 January 2015.

[2] WHO (2012) Accelerating work to overcome the global impact of neglected tropical diseases—a roadmap for implementation. WHO/HTM/NTD/2012.1. http://whqlibdoc.who.int/hq/2012/WHO_HTM_NTD_2012.1_eng.pdf Accessed on 25 January 2015.

[3] UNITING TO COMBAT NTDs (2012) London Declaration on neglected tropical diseases. http://unitingtocombatntds.org/sites/default/files/resource_file/london_declaration_on_ntds.pdf Accessed on 23 May 2014.

[4] WHO (2013) Sustaining the drive to overcome the global impact of neglected tropical diseases—second WHO report on neglected tropical diseases WHO/HTM/NTD/2013.1. Geneva: WHO http://www.who.int/iris/bitstream/10665/77950/1/9789241564540_eng.pdf Accessed on 25 January 2015.

[5] UNITING TO COMBAT NTDs (2014) Delivering on promises and driving progress. http://unitingtocombatntds.org/resource/delivering-promises-and-driving-progress-second-progress-report Accessed on 25 January 2015.

[6] WHO (2013) APOC—status of onchocerciasis in APOC countries. http://www.who.int/apoc/onchocerciasis/status/en/index.html Accessed on 25 January 2015.

[7] Crump A, Morel CM, Omura S (2012) The onchocerciasis chronicle: from the beginning to the end? Trends Parasitol 28: 280–288. S1471-4922(12)00069-4 10.1016/j.pt.2012.04.005 22633470

[8] WHO/APOC (2010) Report of the external mid-term evaluation of the African Programme for Onchocerciasis Control. JAF16.8. http://www.who.int/apoc/MidtermEvaluation_29Oct2010_final_printed.pdf Accessed on 25 January 2015.

[9] WHO (2014) Neglected Tropical Diseases—WHO declares Ecuador free of onchocerciasis (river blindness). http://www.who.int/neglected_diseases/ecuador_free_from_onchocerciasis/en/ Accessed on 14 October 2014.

[10] WHO (2013) Progress towards eliminating onchocerciasis in the WHO Region of the Americas: verification by WHO of elimination of transmission in Colombia. Wkly Epidemiol Rec 88: 381–385. 24052954

[11] The Carter Center (2014) Current transmission status in the 13 foci of the Americas—geographic distribution of onchocerciasis in the Americas. http://www.cartercenter.org/resources/pdfs/health/river_blindness/OEPA-foci.pdf Accessed on 14 October 2014.

[12] PAHO/WHO (2013) 52nd Directing Council (65th session of the regional committee, 30 September-4 October 2013, Washington DC, USA)—towards the elimination of onchocerciasis (river blindness) in the Americas. CD52/INF/4. Pan American Health Organization/World Health Organization. http://www.paho.org/hq/index.php?option=com_docman&task=doc_view&gid=24390&Itemid= Accessed on 25 January 2015.

[13] WHO (2011) 64th World Health Assembly (16–24 May 2011, Geneva, Switzerland)—progress report: onchocerciasis control through ivermectin distribution. A64/26. WHO. http://apps.who.int/gb/ebwha/pdf_files/WHA64/A64_26-en.pdf Accessed on 25 January 2015.

[14] Diawara L, Traoré MO, Badji A, Bissan Y, Doumbia K, Goita SF, Konaté L, Mounkoro K, Sarr MD, Seck AF, Toé L, Tourée S, Remme JHF (2009) Feasibility of onchocerciasis elimination with ivermectin treatment in endemic foci in Africa: first evidence from studies in Mali and Senegal. PLoS Negl Trop Dis 3: e497 10.1371/journal.pntd.0000497 19621091 PMC2710500

[15] Traoré MO, Sarr MD, Badji A, Bissan Y, Diawara L, Doumbia K, Goita SF, Konaté L, Mounkoro K, Seck AF, Toé L, Toure S, Remme JHF (2012) Proof-of-principle of onchocerciasis elimination with ivermectin treatment in endemic foci in Africa: final results of a study in Mali and Senegal. PLoS Negl Trop Dis 6: e1825 10.1371/journal.pntd.0001825 23029586 PMC3441490

[16] Ernst Strüngmann Forum (2010) Disease eradication in the context of global health in the 21st century (August 29-September 3, 2010, Frankfurt am Main, Germany). http://www.esforum.de/forums/esf07_disease_eradication.html Accessed on 20 May 2014.

[17] Damian Walker, Julia Lupp (2013) Guide to preparing an eradication investment case. http://eic-guidelines.org/Guide%20to%20Preparing%20an%20EIC_Draft%208.pdf Accessed on 25 January 2015.

[18] Tediosi F, Steinmann P, de Savigny D, Tanner M (2013) Developing eradication investment cases for onchocerciasis, lymphatic filariasis, and human African trypanosomiasis: rationale and main challenges. PLoS Negl Trop Dis 7: e2446 10.1371/journal.pntd.0002446 24244762 PMC3820723

[19] WHO (2012) Report of the WHO Strategic and Technical Advisory Group for Neglected Tropical Diseases (24–25 April 2012, Geneva, Switzerland). WHO. http://www.who.int/entity/neglected_diseases/NTD_STAG_Report_2012.pdf Accessed on 25 January 2015.

[20] The Carter Center (2013) Fighting disease: Ethiopia—eliminating river blindness. http://www.cartercenter.org/countries/ethiopia-health-river-blindness.html Accessed on 20 April 2014.

[21] Uganda Ministry of Health (2010) Health Sector Strategic Plan III, 2010/11-2014/15. http://www.health.go.ug/docs/HSSP_III_2010.pdf Accessed on 25 January 2015.

[22] Plaisier AP, van Oortmarssen GJ, Habbema JD, Remme J, Alley ES (1990) ONCHOSIM: a model and computer simulation program for the transmission and control of onchocerciasis. Comput Methods Programs Biomed 31: 43–56. 2311368 10.1016/0169-2607(90)90030-d

[23] WHO (2008) APOC—community-directed treatment with ivermectin (CDTI) projects. http://www.who.int/apoc/cdti/projects/en/ Accessed on 23 May 2014.

[24] Noma M, Zoure HG, Tekle AH, Enyong PA, Nwoke BE, Remme JH (2014) The geographic distribution of onchocerciasis in the 20 participating countries of the African Programme for Onchocerciasis Control: (1) priority areas for ivermectin treatment. Parasit Vectors 7: 325 10.1186/1756-3305-7-325 25053266 PMC4223657

[25] UN (2013) Population growth rates. World Population Prospects: the 2012 revision. http://esa.un.org/wpp/Excel-Data/population.htm Accessed on 23 May 2014.

[26] WHO/APOC (2011) Report of the CSA advisory group on onchocerciasis elimination—Annex 1. Predicting the end date of ivermectin treatment in APOC projects. JAF17.7(ii). WHO/APOC.

[27] Coffeng LE, Pion SD, O'Hanlon S, Cousens S, Abiose AO, Fischer PU, Remme JH, Dadzie KY, Murdoch ME, de Vlas SJ, Basanez MG, Stolk WA, Boussinesq M (2013) Onchocerciasis: the pre-control association between prevalence of palpable nodules and skin microfilariae. PLoS Negl Trop Dis 7: e2168 10.1371/journal.pntd.0002168 23593528 PMC3623701

[28] WHO (2014) APOC—community-directed distributor (CDDs). http://www.who.int/apoc/cdti/cdds/en/ Accessed on 20 October 2014.

[29] Omura S (2008) Ivermectin: 25 years and still going strong. Int J Antimicrob Agents 31: 91–98. 18037274 10.1016/j.ijantimicag.2007.08.023

[30] William R.Brieger (2000) Implementation and sustainability of Community-Directed Treatment of Onchocerciasis with ivermectin: a multicountry report. TDR/AFT/RP/96.1. Geneva:UNICEF/UNDP/World Bank/WHO Special Programme for Research and Training in Tropical Diseases.

[31] WHO (2014) APOC—how community-directed treatment with ivermectin (CDTI) began. http://www.who.int/apoc/cdti/history/en/ Accessed on 20 October 2014.

[32] Strebel P, Ottesen EA, Quadros CA, Guirguis S, Hall RG, et al (2011) Group Report: assessing the feasibility of an eradication initiative In: Cochi Stephen L., Dowdle Walter R., editors. Disease eradication in the 21st century: implications for global health. Cambridge, MA: The MIT Press.

[33] WHO/APOC (2007) Revitalising health care delivery in sub-Saharan Africa: the potential of community-directed interventions to strengthen health systems. http://www.who.int/apoc/publications/EN_HealthCare07_7_3_08.pdf Accessed on 25 January 2015.

[34] WHO/APOC (2014) 19th session of Joint Action Forum (11–13 December 2013, Brazzaville, Congo)—Final communique. WHO/APOC. http://www.who.int/entity/apoc/about/structure/jaf/Final_Communique_JAF19_Final_English_140114.pdf Accessed 20 April 2014.

[35] WHO/APOC (2012) 18th session of the Joint Action Forum (Bujumbura, Burundi, 11–13 December 2012)—Final communique. WHO/APOC. http://www.who.int/entity/apoc/about/structure/jaf/Final_Communique_JAF_18_English_final_with_annexes.pdf?ua=1 Accessed on 25 January 2015.

[36] Alley ES, Plaisier AP, Boatin BA, Dadzie KY, Remme J, Zerbo G, Samba EM (1994) The impact of five years of annual ivermectin treatment on skin microfilarial loads in the onchocerciasis focus of Asubende, Ghana. Trans R Soc Trop Med Hyg 88: 581–584. 7992347 10.1016/0035-9203(94)90172-4

[37] Plaisier AP, Alley ES, Boatin BA, van Oortmarssen GJ, Remme H, de Vlas SJ, Bonneux L, Habbema JD (1995) Irreversible effects of ivermectin on adult parasites in onchocerciasis patients in the Onchocerciasis Control Programme in West Africa. J Infect Dis 172: 204–210. 7797912 10.1093/infdis/172.1.204

[38] Coffeng LE, Stolk WA, Hoerauf A, Habbema D, Bakker R, Hopkins AD, de Vlas SJ (2014) Elimination of african onchocerciasis: modeling the impact of increasing the frequency of ivermectin mass treatment. PLoS One 9: e115886 10.1371/journal.pone.0115886 25545677 PMC4278850

[39] WHO/APOC (2010) Conceptual and operational framework of onchocerciasis elimination with ivermectin treatment. WHO/APOC/MG/10.1. http://www.who.int/apoc/oncho_elimination_report_english.pdf Accessed on 25 January 2015.

[40] OEPA (2011) Guide for the detection of a potential recrudescence during the period of Post Treatment Surveillance (PTS). http://www.oepa.net/Documentos/GuiaVEPT/Guide_Detection_Potential_Recrudescence_During_PTS_Englishversion.pdf Accessed on 25 January 2015.

[41] Merck (2011) 2011 Corporate Responsibility Report. http://www.merckresponsibility.com/downloads/MRK_Report_Builder_Full_120824.pdf Accessed on 25 January 2015.

[42] WHO/APOC (2013) The World Health Organization Year 2013 Progress Report. JAF19.5. http://www.who.int/entity/apoc/publications/JAF195_EN_APOC_PR2013_OK.pdf?ua=1 Accessed on 25 January 2015.

[43] Tamarozzi F, Tendongfor N, Enyong PA, Esum M, Faragher B, Wanji S, Taylor MJ (2012) Long term impact of large scale community-directed delivery of doxycycline for the treatment of onchocerciasis. Parasit Vectors 5: 53 10.1186/1756-3305-5-53 22433114 PMC3350421

[44] Tabi TE, Befidi-Mengue R, Nutman TB, Horton J, Folefack A, Pensia E, Fualem R, Fogako J, Gwanmesia P, Quakyi I, Leke R (2004) Human loiasis in a Cameroonian village: a double-blind, placebo-controlled, crossover clinical trial of a three-day albendazole regimen. Am J Trop Med Hyg 71: 211–215. 15306713

[45] WHO (2014) Neglected Tropical Diseases—PCT databank: lymphatic filariasis. http://www.who.int/neglected_diseases/preventive_chemotherapy/lf/en/ Accessed on 29 September 2014.

[46] Bourguinat C, Pion SD, Kamgno J, Gardon J, Duke BO, Boussinesq M, Prichard RK (2007) Genetic selection of low fertile Onchocerca volvulus by ivermectin treatment. PLoS Negl Trop Dis 1: e72 17989786 10.1371/journal.pntd.0000072 PMC2041821

[47] Walker M, Specht S, Churcher TS, Hoerauf A, Taylor MJ, Basanez MG (2014) Therapeutic efficacy and macrofilaricidal activity of doxycycline for the treatment of river blindness. Clin Infect Dis. ciu1152 [pii]. Epub ahead of print. 10.1093/cid/ciu1152 PMC4370165 25537873

[48] DNDi (2014) Bayer and DNDi sign first agreement to develop an innovative oral treatment for human river blindness. 9 December 2014. http://www.dndi.org/media-centre/press-releases/2040-pr-bayer-filaria.html Accessed on 17 January 2014.

[49] Awadzi K, Opoku NO, Attah SK, Lazdins-Helds J, Kuesel AC (2014) A randomized, single-ascending-dose, ivermectin-controlled, double-blind study of moxidectin in Onchocerca volvulus infection. PLoS Negl Trop Dis 8: e2953 10.1371/journal.pntd.0002953 24968000 PMC4072596

[50] Mackenzie CD, Geary TG (2011) Flubendazole: a candidate macrofilaricide for lymphatic filariasis and onchocerciasis field programs. Expert Rev Anti Infect Ther 9: 497–501. 10.1586/eri.11.30 21609260

[51] Udall DN (2007) Recent updates on onchocerciasis: diagnosis and treatment. Clin Infect Dis 44: 53–60. 17143815 10.1086/509325

[52] Lipner EM, Dembele N, Souleymane S, Alley WS, Prevots DR, Toe L, Boatin B, Weil GJ, Nutman TB (2006) Field applicability of a rapid-format anti-Ov-16 antibody test for the assessment of onchocerciasis control measures in regions of endemicity. J Infect Dis 194: 216–221. 16779728 10.1086/505081

[53] Klepac P, Metcalf CJ, McLean AR, Hampson K (2013) Towards the endgame and beyond: complexities and challenges for the elimination of infectious diseases. Philos Trans R Soc Lond B Biol Sci 368: 20120137 10.1098/rstb.2012.0137 23798686 PMC3720036

[54] WHO/APOC (2006) 12th session of the Joint Action Forum (5–8 December 2006, Dar-es-Salaam, Tanzania). JAF12.9. WHO/APOC. http://siteresources.worldbank.org/EXTGLOREGPARPROG/Resources/apoc_so.pdf Accessed on 23 May 2014.

[55] WHO/APOC (2012) Strategic Plan of Action and Budget 2016–2025 for elimination of onchocerciasis in Africa. JAF18.9 (ii). Ouagadougou: WHO/APOC. http://www.who.int/apoc/en_apoc_strategic_plan_2013_ok.pdf Accessed on 25 January 2015.

[56] WHO/APOC (2013) Concept note: transforming APOC into a new regional entity for oncho & LF elimination and support to other PC/NTD. JAF 19.7. Ouagadougou: WHO/APOC. www.who.int/apoc/en_concept_note_ok.pdf Accessed on 25 January 2015.

