# Correction: Control, Elimination, and Eradication of River Blindness: Scenarios, Timelines, and Ivermectin Treatment Needs in Africa

**DOI:** 10.1371/journal.pntd.0003777

**Published:** 2015-05-07

**Authors:** Young Eun Kim, Jan H. F. Remme, Peter Steinmann, Wilma A. Stolk, Jean-Baptiste Roungou, Fabrizio Tediosi

There is an error in the antepenultimate sentence of the abstract. The number of treatments is incorrect. The correct sentence is: The control scenario requires community-directed treatment with ivermectin beyond 2045 with around 2.63 billion treatments over 2013–2045; the elimination scenario, until 2028 in areas where feasible, but beyond 2045 in countries with operational challenges, around 1.48 billion treatments; and the eradication scenario, lasting until 2040, around 1.30 billion treatments.
