# Supplementary material for: Control, Elimination, and Eradication of River Blindness: Scenarios, Timelines, and Ivermectin Treatment Needs in Africa
Source: PLoS Negl Trop Dis. 2015 Apr 10;9(4):e0003664. doi: 10.1371/journal.pntd.0003664 (PMC4393239; doi:10.1371/journal.pntd.0003664)
Supplement: S1 Table — (PDF) [file pntd.0003664.s001.pdf]

**S1 Table. Population, onchocerciasis endemicity, feasibility concern for community-directed treatment with ivermectin (CDTi), start year and frequency of CDTi, treatment coverage, and predicted end year of CDTi for ongoing (as of November 2013) and potential new projects**

| Country | Project | APOC/ former OCP | Population 2014[1] | Pre-control endemicity[2] | Feasibility concern | CDTi start year[3] | CDTi frequency per annum[4] | Treatment coverage[5] | CDTi end year | | |
|---|---|---|---|---|---|---|---|---|---|---|---|
| | | | | | | | | | Control scenario[6] | Elimination scenario[7] | Eradication scenario |
| Angola | Bengo | APOC | 26,658 | Hypo | None | 2010 | 1 | 75% | 2034-2059 | 2018 | 2018 |
| Angola | Benguela | APOC | 50,992 | Hypo | None | 2012 | 1 | 67% | 2036-2061 | 2020 | 2020 |
| Angola | Cuanza Norte | APOC | 26,058 | Hypo | None | 2011 | 1 | 67% | 2035-2060 | 2017 | 2017 |
| Angola | Huila | APOC | 239,930 | Meso | None | 2010 | 1 | 70% | 2034-2059 | 2018 | 2018 |
| Angola | Kuando Kubango | APOC | 413,646 | Meso | None | 2009 | 1 | 76% | 2033-2058 | 2015 | 2015 |
| Angola | Lunda Norte | APOC | 309,045 | Meso | None | 2009 | 1 | 62% | 2033-2058 | 2017 | 2017 |
| Angola | Lunda sul | APOC | 260,377 | Meso | None | 2009 | 1 | 69% | 2033-2058 | 2017 | 2017 |
| Angola | Moxico 1 | APOC | 272,116 | Meso | None | 2011 | 1 | 67% | 2035-2060 | 2018 | 2018 |
| Angola | NY Benguela | APOC | 114,544 | Hyper | None | 2014 | 1 | 67% | Not targeted | 2025 | 2025 |
| Angola | NY Cuanza Norte | APOC | 18,688 | Hypo | None | 2014 | 1 | 67% | Not targeted | 2022 | 2022 |
| Angola | NY Huila | APOC | 23,189 | Meso | None | 2014 | 1 | 67% | Not targeted | 2022 | 2022 |
| Angola | NY Lunda Norte | APOC | 67,327 | Hypo | None | 2014 | 1 | 67% | Not targeted | 2022 | 2022 |

[1] 2012 population (source: APOC treatment database) was adjusted for population growth rates (UN (2013) World Population Prospects: The 2012 Revision. http://esa.un.org/wpp/Excel-Data/population.htm Accessed on 23 May 2014)

[2] APOC countries: non-endemic with highest nodule prevalence in adult males < 5%, hypo-endemic between 5% and 20%, meso-endemic between 20% and 40%, and hyper-endemic with 40% and above

Former OCP countries: non-endemic with <10% microfilariae prevalence in ages 5+, hypo-endemic between 10% to 40%, meso-endemic between 40% and 60%, and hyper-endemic with 60% and above

[3] Ongoing projects (as of November 2013): The first year with treatment coverage greater than 60% was used, as effective control of the disease requires the treatment coverage of 60% and above. New projects: The start year was predicted based on APOC's strategic plan to focus on the onchocerciasis elimination for the next decade 2016-2025, the current epidemiology, and the current political situation.

[4] Six-monthly: only new projects in Ethiopia and Uganda where respective ministries of health announced six-monthly drug administration in new projects to bring them in line with ongoing projects

[5] APOC countries: average over 2010-2012 at project/country/regional levels, former OCP countries: latest available data or average at country level

[6] The lower bound is the expected end year without the assumption of possible CDTi extension for another 25 years due to insufficient treatment coverage, and the upper bound with the assumption. This assumption was applied to APOC countries except Equatorial Guinea that is known to have almost eliminated the disease and Mozambique that is endemic in limited border areas where less than 70,000 people live. The former OCP countries were also excluded from this assumption considering the level of transmission has decreased significantly due to CDTi implemented since the 1990s and the recent regional treatment coverage was around 80% on average.

[7] The control scenario was applied to projects with feasibility concerns. For these projects, the lower bound of the CDTi end year was expected without the assumption of possible CDTi extension for another 25 years due to insufficient treatment coverage, and the upper bound with the assumption.

| Country | Project | APOC/ former OCP | Population 2014[1] | Pre-control endemicity[2] | Feasibility concern | CDTi start year[3] | CDTi frequency per annum[4] | Treatment coverage[5] | CDTi end year | | |
|---|---|---|---|---|---|---|---|---|---|---|---|
| | | | | | | | | | Control scenario[6] | Elimination scenario[7] | Eradication scenario |
| Angola | NY Moxico 1 | APOC | 347,350 | Hypo | None | 2014 | 1 | 67% | Not targeted | 2022 | 2022 |
| Angola | P5Angola | APOC | 264,252 | Hypo | None | 2015 | 1 | 67% | Not targeted | 2023 | 2023 |
| Angola | Uige | APOC | 191,005 | Meso | None | 2014 | 1 | 67% | Not targeted | 2023 | 2023 |
| Angola | Zaire | APOC | 14,407 | Hypo | None | 2014 | 1 | 67% | Not targeted | 2021 | 2021 |
| Benin | Benin | former OCP | 3,585,280 | Hyper | None | 1990s | 1 | 48% | 2016 | 2016 | 2016 |
| Burkina Faso | BF Bougouriba | former OCP | 77,626 | Hyper | None | 1990s | 1 | 84% | 2016 | 2016 | 2016 |
| Burkina Faso | BF Leraba Comoe | former OCP | 152,125 | Hyper | None | 2010 | 1 | 84% | 2034 | 2021 | 2021 |
| Burundi | Bururi | APOC | 387,223 | Meso | None | 2008 | 1 | 75% | 2032-2057 | 2014 | 2014 |
| Burundi | Cibitoke-Bubanza | APOC | 924,648 | Meso | None | 2006 | 1 | 79% | 2030-2055 | 2014 | 2014 |
| Burundi | Rutana | APOC | 301,453 | Hypo | None | 2008 | 1 | 77% | 2032-2057 | 2016 | 2016 |
| Cameroon | Adamaoua 1 | APOC | 506,733 | Hyper | None | 2008 | 1 | 74% | 2032-2057 | 2021 | 2021 |
| Cameroon | Adamaoua 2 | APOC | 460,444 | Hyper | None | 2004 | 1 | 77% | 2028-2053 | 2016 | 2016 |
| Cameroon | Centre 1 | APOC | 467,973 | Hyper | None | 2003 | 1 | 78% | 2027-2052 | 2024 | 2024 |
| Cameroon | Centre 2 | APOC | 110,151 | Hyper | None | 2005 | 1 | 77% | 2029-2054 | 2018 | 2018 |
| Cameroon | Centre 3 | APOC | 355,666 | Hyper | None | 2004 | 1 | 77% | 2028-2053 | 2017 | 2017 |
| Cameroon | East | APOC | 129,904 | Hyper | None | 2007 | 1 | 78% | 2031-2056 | 2018 | 2018 |
| Cameroon | Far North | APOC | 307,244 | Meso | None | 2007 | 1 | 80% | 2031-2056 | 2016 | 2016 |
| Cameroon | Littoral 1 | APOC | 307,171 | Hyper | None | 2007 | 1 | 78% | 2031-2056 | 2026 | 2026 |
| Cameroon | Littoral 2 | APOC | 163,866 | Hyper | None | 2003 | 1 | 78% | 2027-2052 | 2020 | 2020 |
| Cameroon | Northern | APOC | 688,951 | Hyper | None | 2004 | 1 | 78% | 2028-2053 | 2021 | 2021 |
| Cameroon | Northwest | APOC | 895,057 | Hyper | None | 2005 | 1 | 78% | 2029-2054 | 2017 | 2017 |
| Cameroon | P20Cameroon | APOC | 164,900 | Hyper | None | 2015 | 1 | 78% | 2039-2064 | 2022 | 2022 |
| Cameroon | P5Cameroon_forest | APOC | 335,977 | Hypo | None | 2014 | 1 | 78% | Not targeted | 2020 | 2020 |
| Cameroon | P5Cameroon_forest_noloa | APOC | 110,356 | Hypo | None | 2014 | 1 | 78% | Not targeted | 2020 | 2020 |
| Cameroon | P5Cameroon_savannah | APOC | 1,194,396 | Hypo | None | 2014 | 1 | 78% | Not targeted | 2020 | 2020 |
| Cameroon | South | APOC | 327,119 | Hyper | None | 2006 | 1 | 78% | 2030-2055 | 2019 | 2019 |
| Cameroon | South West 1 | APOC | 428,877 | Hyper | None | 2005 | 1 | 78% | 2029-2054 | 2020 | 2020 |
| Cameroon | South West 2 | APOC | 291,874 | Hyper | None | 2004 | 1 | 78% | 2028-2053 | 2019 | 2019 |
| Cameroon | Western | APOC | 1,793,241 | Hyper | None | 2003 | 1 | 80% | 2027-2052 | 2024 | 2024 |

| Country | Project | APOC/ former OCP | Population 2014[1] | Pre-control endemicity[2] | Feasibility concern | CDTi start year[3] | CDTi frequency per annum[4] | Treatment coverage[5] | CDTi end year | | |
|---|---|---|---|---|---|---|---|---|---|---|---|
| | | | | | | | | | Control scenario[6] | Elimination scenario[7] | Eradication scenario |
| CAR[8] | CAR combined project | APOC | 1,943,659 | Hyper | (post) conflict | 2003 | 1 | 80% | 2027-2052 | 2027-2052 | 2024 |
| CAR | P20CAR | APOC | 63,224 | Hyper | (post) conflict | 2016 | 1 | 80% | 2040-2065 | 2040-2065 | 2034 |
| CAR | P5CAR | APOC | 143,609 | Hypo | (post) conflict | 2020 | 1 | 80% | Not targeted | Not targeted | 2026 |
| Chad | Chad | APOC | 2,181,933 | Hyper | None | 2001 | 1 | 81% | 2025-2050 | 2015 | 2015 |
| Congo | Congo 1 | APOC | 904,556 | Hyper | None | 2007 | 1 | 81% | 2031-2056 | 2016 | 2016 |
| Congo | P20Congo | APOC | 39,517 | Hyper | None | 2014 | 1 | 81% | 2038-2063 | 2026 | 2026 |
| Congo | P5Congo | APOC | 530,726 | Hypo | None | 2014 | 1 | 81% | Not targeted | 2020 | 2020 |
| Côte d’Ivoire | Bandama | former OCP | 1,008,980 | Hyper | None | 1990s | 1 | 73% | 2020 | 2020 | 2020 |
| Côte d`Ivoire | CI Comoe | former OCP | 622,485 | Hyper | None | 1990s | 1 | 73% | 2035 | 2023 | 2023 |
| Côte d`Ivoire | CI Lower Sassandra | former OCP | 350,025 | Hyper | None | 2014 | 1 | 73% | 2038 | 2028 | 2028 |
| Côte d`Ivoire | CI Upper Sassandra | former OCP | 377,662 | Hyper | None | 1990s | 1 | 73% | 2020 | 2020 | 2020 |
| DRC[9] | Bandundu | APOC | 6,436 | Hyper | (post) conflict | 2005 | 1 | 82% | 2029-2054 | 2029-2054 | 2021 |
| DRC | Bas-Congo Kinshasa | APOC | 1,523,869 | Hyper | (post) conflict | 2008 | 1 | 71% | 2032-2057 | 2032-2057 | 2023 |
| DRC | Butembo-Beni | APOC | 946,872 | Hyper | (post) conflict | 2011 | 1 | 56% | 2035-2060 | 2035-2060 | 2031 |
| DRC | Equateur-Kiri | APOC | 1,258,400 | Hyper | (post) conflict | 2009 | 1 | 79% | 2033-2058 | 2033-2058 | 2022 |
| DRC | Ituri-Nord | APOC | 1,273,305 | Hyper | (post) conflict | 2009 | 1 | 71% | 2033-2058 | 2033-2058 | 2030 |
| DRC | Ituri-Sud | APOC | 1,163,935 | Hyper | (post) conflict | 2012 | 1 | 71% | 2036-2061 | 2036-2061 | 2036 |
| DRC | Kasai | APOC | 10,917,310 | Hyper | None | 2009 | 1 | 75% | 2033-2058 | 2031 | 2031 |
| DRC | Kasongo | APOC | 1,367,616 | Hyper | (post) conflict | 2009 | 1 | 71% | 2033-2058 | 2033-2058 | 2022 |
| DRC | Katanga-Nord | APOC | 635,388 | Hyper | (post) conflict | 2009 | 1 | 71% | 2033-2058 | 2033-2058 | 2030 |
| DRC | Katanga-Sud | APOC | 703,780 | Hyper | (post) conflict | 2009 | 1 | 71% | 2033-2058 | 2033-2058 | 2030 |
| DRC | Lualaba | APOC | 228,284 | Hyper | (post) conflict | 2008 | 1 | 80% | 2032-2057 | 2032-2057 | 2024 |
| DRC | Lubutu | APOC | 339,104 | Hyper | (post) conflict | 2009 | 1 | 61% | 2033-2058 | 2033-2058 | 2028 |
| DRC | Masisi-Walikale | APOC | 1,062,822 | Hyper | (post) conflict | 2010 | 1 | 71% | 2034-2059 | 2034-2059 | 2032 |
| DRC | Mongala | APOC | 1,477,897 | Hyper | (post) conflict | 2009 | 1 | 78% | 2033-2058 | 2033-2058 | 2030 |
| DRC | NY Katanga-Nord | APOC | 461,048 | Meso | (post) conflict | 2014 | 1 | 71% | Not targeted | 2038-2063 | 2021 |
| DRC | NY Lualaba | APOC | 997,775 | Hyper | (post) conflict | 2014 | 1 | 71% | Not targeted | 2038-2063 | 2031 |

[8] Central African Republic
[9] Democratic Republic of the Congo

| Country | Project | APOC/ former OCP | Population 2014[1] | Pre-control endemicity[2] | Feasibility concern | CDTi start year[3] | CDTi frequency per annum[4] | Treatment coverage[5] | CDTi end year | | |
|---|---|---|---|---|---|---|---|---|---|---|---|
| | | | | | | | | | Control scenario[6] | Elimination scenario[7] | Eradication scenario |
| DRC | NY Masisi-Walikale | APOC | 55,387 | Hyper | (post) conflict | 2014 | 1 | 71% | 2038-2063 | 2038-2063 | 2027 |
| DRC | NY Rutshuru-Ngoma | APOC | 8,621 | Meso | (post) conflict | 2014 | 1 | 71% | 2038-2063 | 2038-2063 | 2023 |
| DRC | NY Sankuru | APOC | 476,028 | Hyper | (post) conflict | 2014 | 1 | 71% | Not targeted | 2038-2063 | 2027 |
| DRC | NY Ueles | APOC | 161,944 | Hyper | (post) conflict | 2014 | 1 | 71% | 2038-2063 | 2038-2063 | 2035 |
| DRC | P20DRC | APOC | 2,387,370 | Hyper | (post) conflict | 2016 | 1 | 71% | 2040-2065 | 2040-2065 | 2037 |
| DRC | P5DRC | APOC | 7,591,705 | Hypo | (post) conflict | 2020 | 1 | 71% | Not targeted | Not targeted | 2027 |
| DRC | Rutshuru-Ngoma | APOC | 669,404 | Hyper | (post) conflict | 2009 | 1 | 74% | 2033-2058 | 2033-2058 | 2018 |
| DRC | Sankuru | APOC | 1,082,968 | Hyper | (post) conflict | 2007 | 1 | 82% | 2031-2056 | 2031-2056 | 2028 |
| DRC | Tshopo | APOC | 1,617,757 | Hyper | (post) conflict | 2010 | 1 | 63% | 2037-2062 | 2037-2062 | 2037 |
| DRC | Tshuapa | APOC | 1,441,333 | Hyper | (post) conflict | 2010 | 1 | 66% | 2034-2059 | 2034-2059 | 2033 |
| DRC | Ubangi-Nord | APOC | 811,591 | Hyper | (post) conflict | 2011 | 1 | 71% | 2037-2062 | 2037-2062 | 2037 |
| DRC | Ubangi-Sud | APOC | 1,368,969 | Hyper | (post) conflict | 2011 | 1 | 71% | 2035-2060 | 2035-2060 | 2023 |
| DRC | Ueles | APOC | 1,596,501 | Hyper | (post) conflict | 2006 | 1 | 71% | 2030-2055 | 2030-2055 | 2028 |
| Equatorial Guinea | Bioko | APOC | 88,252 | Hyper | None | 2007 | 1 | 71% | 2031 | 2020 | 2020 |
| Ethiopia | Assosa | APOC | 579,207 | Meso | None | 2014 | 2 | 79% | Not targeted | 2021 | 2021 |
| Ethiopia | Bench-Maji | APOC | 765,856 | Hyper | None | 2005 | 1 | 79% | 2029-2054 | 2017 | 2017 |
| Ethiopia | East Wellega | APOC | 937,884 | Meso | None | 2006 | 1 | 79% | 2030-2055 | 2015 | 2015 |
| Ethiopia | Gambella | APOC | 112,052 | Hyper | None | 2006 | 1 | 78% | 2030-2055 | 2015 | 2015 |
| Ethiopia | Horo Guduru | APOC | 54,995 | Meso | None | 2014 | 2 | 79% | Not targeted | 2021 | 2021 |
| Ethiopia | Illubabor | APOC | 793,275 | Hyper | None | 2004 | 1 | 82% | 2028-2053 | 2017 | 2017 |
| Ethiopia | Jimma | APOC | 936,393 | Meso | None | 2004 | 1 | 83% | 2028-2053 | 2015 | 2015 |
| Ethiopia | Kaffa-Sheka | APOC | 1,329,915 | Hyper | None | 2003 | 1 | 79% | 2027-2052 | 2015 | 2015 |
| Ethiopia | Kamashi | APOC | 494,563 | Hyper | None | 2014 | 2 | 79% | 2038-2063 | 2023 | 2023 |
| Ethiopia | Metekel | APOC | 169,262 | Meso | None | 2007 | 1 | 74% | 2031-2056 | 2015 | 2015 |
| Ethiopia | North Gondar | APOC | 328,659 | Meso | None | 2004 | 1 | 74% | 2028-2053 | 2015 | 2015 |
| Ethiopia | NY East Wellega | APOC | 300,211 | Hyper | None | 2014 | 2 | 79% | 2038-2063 | 2022 | 2022 |
| Ethiopia | NY West Wellega | APOC | 304,914 | Meso | None | 2014 | 2 | 79% | Not targeted | 2021 | 2021 |
| Ethiopia | P20Ethiopia | APOC | 348,726 | Hyper | None | 2014 | 2 | 79% | 2038-2063 | 2022 | 2022 |
| Ethiopia | P5Ethiopia | APOC | 3,682,280 | Hypo | None | 2014 | 2 | 79% | Not targeted | 2020 | 2020 |

| Country | Project | APOC/ former OCP | Population 2014[1] | Pre-control endemicity[2] | Feasibility concern | CDTi start year[3] | CDTi frequency per annum[4] | Treatment coverage[5] | CDTi end year: Control scenario[6] | CDTi end year: Elimination scenario[7] | CDTi end year: Eradication scenario |
|---|---|---|---|---|---|---|---|---|---|---|---|
| Ethiopia | West Shewa | APOC | 60,625 | Hypo | None | 2014 | 2 | 79% | Not targeted | 2019 | 2019 |
| Ethiopia | West Wellega | APOC | 1,076,961 | Hyper | None | 2006 | 1 | 79% | 2030-2055 | 2015 | 2015 |
| Gabon | P5Gabon | APOC | 85,468 | Hypo | *Loa loa* coendemicity | 2021 | 1 | 76% | Not targeted | Not targeted | 2027 |
| Ghana | Ghana Center | former OCP | 91,065 | Hyper | None | 1990s | 1 | 77% | 2020 | 2020 | 2020 |
| Ghana | Ghana North | former OCP | 1,229,326 | Hyper | None | 1990s | 1 | 77% | 2018 | 2018 | 2018 |
| Ghana | Ghana South CI border | former OCP | 709,561 | Hypo | None | 2010 | 1 | 77% | 2034 | 2015 | 2015 |
| Ghana | Ghana South Rx | former OCP | 431,415 | Hypo | None | 2010 | 1 | 77% | 2034 | 2015 | 2015 |
| Ghana | Ghana Tano new Rx | former OCP | 74,115 | Hypo | None | 2015 | 1 | 77% | 2039 | 2020 | 2020 |
| Guinea | Guinea border | former OCP | 2,384,879 | Hyper | None | 1990s | 1 | 81% | 2020 | 2020 | 2020 |
| Guinea | Guinea Main | former OCP | 946,807 | Hyper | None | 1990s | 1 | 81% | 2016 | 2016 | 2016 |
| Guinea-Bissau | Guinea Bissau | former OCP | 194,915 | Hyper | None | 1990s | 1 | 75% | 2016 | 2016 | 2016 |
| Liberia | Northwestern | APOC | 1,878,803 | Meso | None | 2009 | 1 | 77% | 2033-2058 | 2016 | 2016 |
| Liberia | Southeastern | APOC | 528,362 | Meso | None | 2006 | 1 | 83% | 2030-2055 | 2016 | 2016 |
| Liberia | Southwestern | APOC | 761,649 | Meso | None | 2007 | 1 | 77% | 2031-2056 | 2014 | 2014 |
| Malawi | Malawi Extension | APOC | 1,282,457 | Meso | None | 2004 | 1 | 82% | 2028-2053 | 2016 | 2016 |
| Malawi | Thyolo Mwanza | APOC | 978,116 | Meso | None | 2004 | 1 | 83% | 2028-2053 | 2015 | 2015 |
| Mali | Mali | former OCP | 5,145,734 | Hyper | None | 1990s | 1 | 82% | 2016 | 2016 | 2016 |
| Mozambique | P20Mozambique | APOC | 17,030 | Meso | None | 2015 | 1 | 76% | 2039 | 2023 | 2023 |
| Mozambique | P5Mozambique | APOC | 49,746 | Hypo | None | 2015 | 1 | 76% | Not targeted | 2022 | 2022 |
| Nigeria | Adamawa | APOC | 1,861,788 | Hyper | None | 2001 | 1 | 80% | 2025-2050 | 2015 | 2015 |
| Nigeria | Akwa Ibom | APOC | 31,875 | Meso | None | 2006 | 1 | 84% | 2030-2055 | 2016 | 2016 |
| Nigeria | Bauchi | APOC | 1,907,526 | Meso | None | 2009 | 1 | 79% | 2033-2058 | 2015 | 2015 |
| Nigeria | Benue | APOC | 3,761,997 | Hyper | None | 2007 | 1 | 77% | 2031-2056 | 2019 | 2019 |
| Nigeria | Borno | APOC | 1,499,554 | Meso | None | 2006 | 1 | 83% | 2030-2055 | 2015 | 2015 |
| Nigeria | Cross River | APOC | 1,359,649 | Hyper | None | 1999 | 1 | 81% | 2023-2048 | 2015 | 2015 |
| Nigeria | Edo Delta | APOC | 1,748,040 | Hyper | None | 1999 | 1 | 80% | 2023-2048 | 2015 | 2015 |
| Nigeria | Ekiti | APOC | 2,359,828 | Meso | None | 2004 | 1 | 76% | 2028-2053 | 2015 | 2015 |
| Nigeria | Enugu Anambra Ebony | APOC | 2,597,471 | Hyper | None | 1999 | 1 | 80% | 2023-2048 | 2015 | 2015 |
| Nigeria | FCT | APOC | 551,432 | Meso | None | 2004 | 1 | 82% | 2028-2053 | 2016 | 2016 |

| Country | Project | APOC/ former OCP | Population 2014[1] | Pre-control endemicity[2] | Feasibility concern | CDTi start year[3] | CDTi frequency per annum[4] | Treatment coverage[5] | CDTi end year | | |
|---|---|---|---|---|---|---|---|---|---|---|---|
| | | | | | | | | | Control scenario[6] | Elimination scenario[7] | Eradication scenario |
| Nigeria | Gombe | APOC | 2,072,712 | Hyper | None | 2006 | 1 | 82% | 2030-2055 | 2017 | 2017 |
| Nigeria | Imo Abia | APOC | 1,431,900 | Hyper | None | 1999 | 1 | 80% | 2023-2048 | 2017 | 2017 |
| Nigeria | Jigawa | APOC | 388,171 | Hypo | None | 2004 | 1 | 74% | 2028-2053 | 2015 | 2015 |
| Nigeria | Kaduna | APOC | 3,016,022 | Meso | None | 2010 | 1 | 80% | 2034-2059 | 2018 | 2018 |
| Nigeria | Kano | APOC | 1,076,195 | Hyper | None | 2000 | 1 | 81% | 2024-2049 | 2016 | 2016 |
| Nigeria | Kebbi | APOC | 226,137 | Hypo | None | 2006 | 1 | 78% | 2030-2055 | 2016 | 2016 |
| Nigeria | Kogi | APOC | 1,943,133 | Hyper | None | 1999 | 1 | 82% | 2023-2048 | 2015 | 2015 |
| Nigeria | Kwara | APOC | 1,642,329 | Hyper | None | 2000 | 1 | 81% | 2024-2049 | 2015 | 2015 |
| Nigeria | Niger | APOC | 2,887,036 | Meso | None | 2004 | 1 | 79% | 2028-2053 | 2015 | 2015 |
| Nigeria | Ogun | APOC | 374,030 | Meso | None | 2003 | 1 | 82% | 2027-2052 | 2015 | 2015 |
| Nigeria | Ondo | APOC | 1,487,782 | Meso | None | 2001 | 1 | 81% | 2025-2050 | 2015 | 2015 |
| Nigeria | Osun | APOC | 1,755,255 | Meso | None | 2009 | 1 | 79% | 2033-2058 | 2016 | 2016 |
| Nigeria | Oyo | APOC | 1,197,059 | Meso | None | 2011 | 1 | 80% | 2035-2060 | 2018 | 2018 |
| Nigeria | P20Nigeria | APOC | 241,777 | Hyper | None | 2014 | 1 | 80% | 2038-2063 | 2026 | 2026 |
| Nigeria | P5Nigeria | APOC | 5,847,463 | Hypo | None | 2014 | 1 | 80% | Not targeted | 2020 | 2020 |
| Nigeria | P5Nigeria | APOC | 2,362,136 | Hypo | None | 2014 | 1 | 80% | Not targeted | 2020 | 2020 |
| Nigeria | Plateau Nassarawa | APOC | 1,664,490 | Meso | None | 2000 | 1 | 82% | 2024-2049 | 2015 | 2015 |
| Nigeria | Plateau Nassarawa LF | APOC | 1,685,205 | Hyper | None | 2000 | 1 | 80% | 2024-2049 | 2015 | 2015 |
| Nigeria | Taraba | APOC | 1,810,936 | Hyper | None | 2009 | 1 | 82% | 2033-2058 | 2020 | 2020 |
| Nigeria | Yobe | APOC | 669,776 | Meso | None | 2002 | 1 | 78% | 2026-2051 | 2015 | 2015 |
| Nigeria | Zamfara | APOC | 310,839 | Hypo | None | 1999 | 1 | 78% | 2023-2048 | 2016 | 2016 |
| Senegal | Senegal | former OCP | 187,405 | Hyper | None | 1990s | 1 | 79% | 2015 | 2015 | 2015 |
| Sierra Leone | Sierra Leone | former OCP | 3,319,643 | Hyper | None | 2008 | 1 | 77% | 2032 | 2021 | 2021 |
| South Sudan | East Bahr El Ghazal | APOC | 619,344 | Hyper | (post) conflict | 2011 | 1 | 60% | 2035-2060 | 2035-2060 | 2020 |
| South Sudan | East Equatoria | APOC | 1,100,863 | Hyper | (post) conflict | 2009 | 1 | 67% | 2033-2058 | 2033-2058 | 2019 |
| South Sudan | P20SouthSudan | APOC | 31,012 | Hyper | (post) conflict | 2017 | 1 | 60% | 2041-2066 | 2041-2066 | 2040 |
| South Sudan | P5SouthSudan | APOC | 853,648 | Hypo | (post) conflict | 2021 | 1 | 60% | Not targeted | Not targeted | 2029 |
| South Sudan | Upper Nile | APOC | 576,858 | Meso | (post) conflict | 2010 | 1 | 56% | 2034-2059 | 2034-2059 | 2018 |
| South Sudan | West Bahr El Ghazal | APOC | 3,338,305 | Hyper | (post) conflict | 2011 | 1 | 60% | 2035-2060 | 2035-2060 | 2035 |

| Country | Project | APOC/ former OCP | Population 2014[1] | Pre-control endemicity[2] | Feasibility concern | CDTi start year[3] | CDTi frequency per annum[4] | Treatment coverage[5] | CDTi end year | | |
|---|---|---|---|---|---|---|---|---|---|---|---|
| | | | | | | | | | Control scenario[6] | Elimination scenario[7] | Eradication scenario |
| South Sudan | West Equatoria | APOC | 787,420 | Hyper | (post) conflict | 2009 | 1 | 71% | 2033-2058 | 2033-2058 | 2026 |
| Sudan | P5Sudan | APOC | 227,384 | Hypo | None | 2014 | 1 | 82% | Not targeted | 2020 | 2020 |
| Sudan | Sudan | APOC | 214,782 | Hypo | None | 2008 | 1 | 82% | 2032-2057 | 2016 | 2016 |
| Sudan | Sudan Abu Hamed | APOC | 214,782 | Hypo | None | 2008 | 1 | 82% | 2032-2057 | 2016 | 2016 |
| Tanzania | Kilosa | APOC | 551,542 | Meso | None | 2004 | 1 | 80% | 2028-2053 | 2015 | 2015 |
| Tanzania | Mahenge | APOC | 551,412 | Hyper | None | 2003 | 1 | 80% | 2027-2052 | 2022 | 2022 |
| Tanzania | Morogoro | APOC | 394,691 | Meso | None | 2006 | 1 | 80% | 2030-2055 | 2015 | 2015 |
| Tanzania | P5Tanzania | APOC | 1,000,102 | Hypo | None | 2015 | 1 | 81% | Not targeted | 2021 | 2021 |
| Tanzania | Ruvuma | APOC | 435,723 | Hyper | None | 2002 | 1 | 81% | 2026-2051 | 2023 | 2023 |
| Tanzania | Tanga | APOC | 341,837 | Meso | None | 2004 | 1 | 81% | 2028-2053 | 2016 | 2016 |
| Tanzania | Tukuyu | APOC | 125,098 | Meso | None | 2001 | 1 | 80% | 2025-2050 | 2015 | 2015 |
| Tanzania | Tunduru | APOC | 135,650 | Hyper | None | 2005 | 1 | 82% | 2029-2054 | 2017 | 2017 |
| Togo | Togo | former OCP | 3,171,784 | Hyper | None | 1992 | 1 | 83% | 2016 | 2016 | 2016 |
| Uganda | P5Uganda | APOC | 271,652 | Hypo | None | 1990s | 2 | 75% | Not targeted | 2022 | 2022 |
| Uganda | Phase 1 | APOC | 425,538 | Hyper | None | 2001 | 1 | 75% | 2025-2050 | 2015 | 2015 |
| Uganda | Phase 2 | APOC | 827,960 | Hypo | None | 2000 | 1 | 75% | 2024-2049 | 2016 | 2016 |
| Uganda | Phase 3 | APOC | 1,527,173 | Hyper | None | 2003 | 1 | 75% | 2027-2052 | 2027 | 2027 |
| Uganda | Phase 4 | APOC | 864,444 | Hyper | None | 1999 | 1 | 75% | 2023-2048 | 2015 | 2015 |
| Uganda | Phase 5 | APOC | 556,618 | Hyper | None | 2012 | 2 | 75% | 2036-2061 | 2023 | 2023 |
