# Supplementary material for: Control, Elimination, and Eradication of River Blindness: Scenarios, Timelines, and Ivermectin Treatment Needs in Africa
Source: PLoS Negl Trop Dis. 2015 Apr 10;9(4):e0003664. doi: 10.1371/journal.pntd.0003664 (PMC4393239; doi:10.1371/journal.pntd.0003664)
Supplement: S2 Table — (PDF) [file pntd.0003664.s002.pdf]

S2 Table. Summary of treatment coverage and distribution parameters for probabilistic sensitivity analysis

Country	Average[1]	Standard deviation	Beta distribution[2]	
			Alpha	Beta
**APOC countries**				
Angola	67%	0.11	12.36	5.97
Burundi	77%	0.04	105.52	31.48
Cameroon	78%	0.05	62.60	17.72
Central African Republic	80%	0.03	167.75	40.98
Chad	81%	0.00	124,658.19	29,240.81
Congo	81%	0.02	207.63	47.39
Democratic Republic of the Congo	71%	0.13	7.47	3.12
Equatorial Guinea	71%	0.00	292,469.03	119,749.47
Ethiopia	79%	0.04	77.58	20.87
Liberia	77%	0.10	13.31	3.89
Malawi	83%	0.00	5,112.61	1,065.77
Nigeria	80%	0.04	83.33	21.00
South Sudan	60%	0.12	8.72	5.78
Sudan	82%	0.03	167.06	37.92
Tanzania	81%	0.01	868.01	210.26
Uganda	75%	0.11	10.48	3.46
**APOC**	**76%**	**0.06**	**35.04**	**10.81**
**Country**	**Latest[3]**	**Standard deviation[4]**	**Beta distribution**	
			**Alpha**	**Beta**
**Former OCP countries**				
Benin	48%	0.05	51.42	55.48
Burkina Faso	84%	0.08	15.56	3.05
Côte d'Ivoire	84%	0.08	15.56	3.05
Ghana	73%	0.07	26.67	10.07
Guinea	73%	0.07	26.67	10.07
Guinea-Bissau	73%	0.07	26.67	10.07
Mali	73%	0.07	26.67	10.07
Senegal	77%	0.08	21.83	6.37
Sierra Leone	80%	0.08	18.90	4.64
Togo	77%	0.08	21.83	6.37
**Former OCP**	**74%**	**0.10**	**13.09**	**4.58**

[1] The average treatment coverage over 2010-2012

[2] Parameters of *Beta(α, β)* were estimated using a method of moments:

$$\hat{\alpha} = \bar{x}\left(\frac{\bar{x}(1-\bar{x})}{\bar{v}} - 1\right), \hat{\beta} = (1-\bar{x})\left(\frac{\bar{x}(1-\bar{x})}{\bar{v}} - 1\right), if\ \bar{v} < \bar{x}(1-\bar{x}),\ where\ \bar{x}\ is\ a\ sample\ mean, \bar{v}\ is\ a\ sample\ variance$$

[3] A provisional database for the former OCP countries had only the latest treatment coverage.

[4] The standard deviation was assumed to be 10% of the treatment coverage.
