# Supplementary figures and images for: Control, Elimination, and Eradication of River Blindness: Scenarios, Timelines, and Ivermectin Treatment Needs in Africa
Source: PLoS Negl Trop Dis. 2015 Apr 10;9(4):e0003664. doi: 10.1371/journal.pntd.0003664 (PMC4393239; doi:10.1371/journal.pntd.0003664)

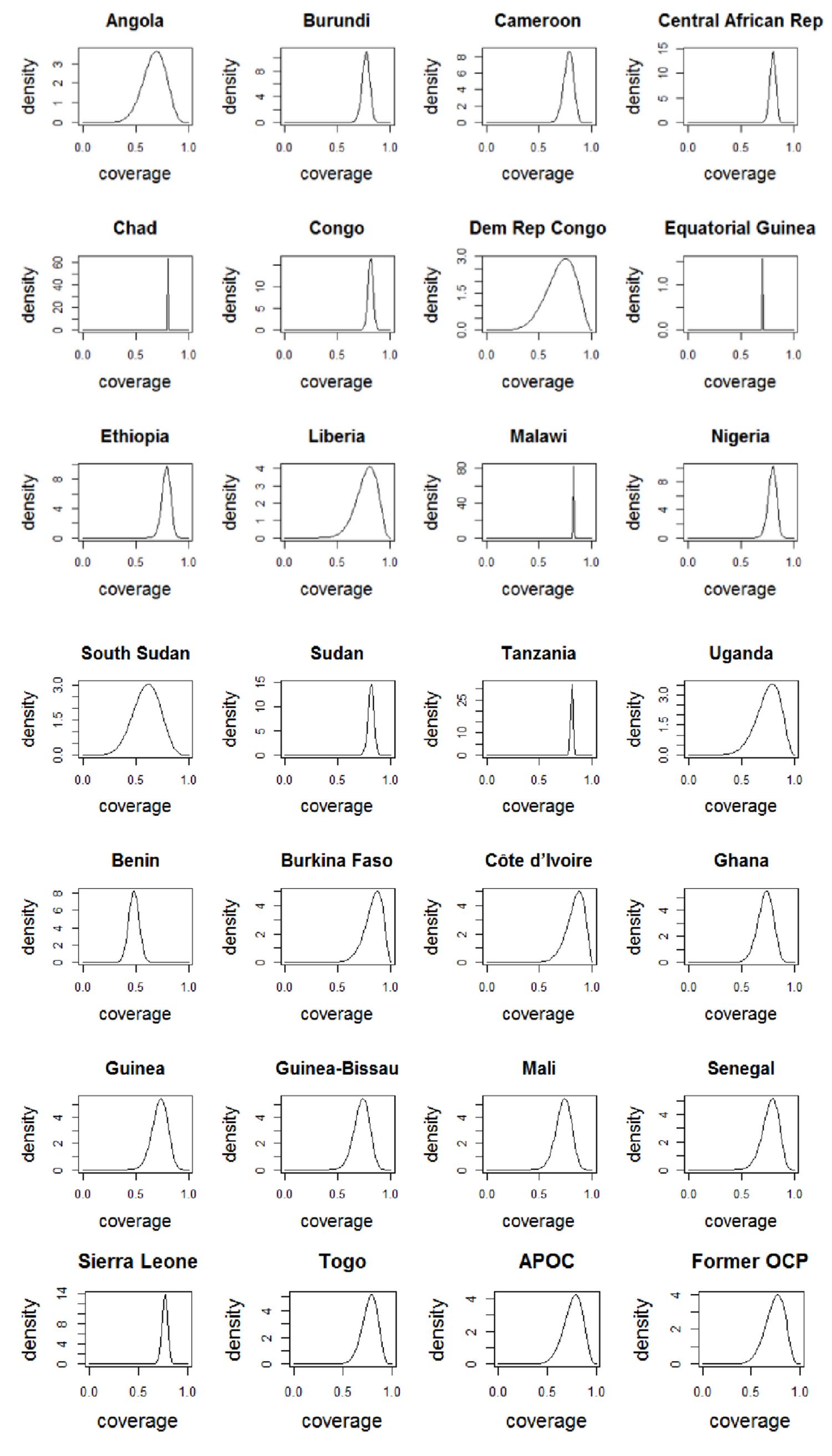

Supplement: S1 Fig — (TIF) [file pntd.0003664.s003.tif]

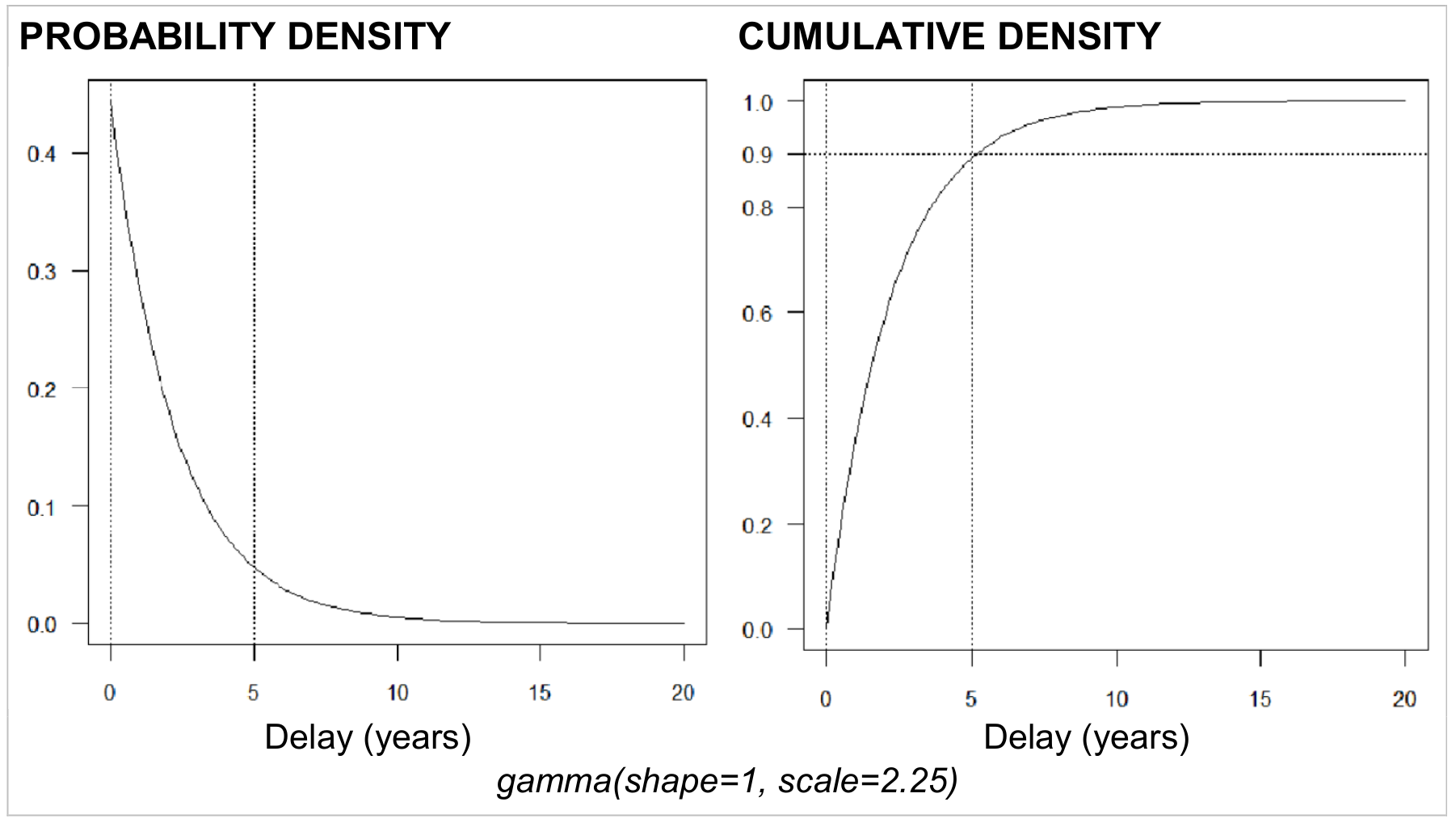

Supplement: S2 Fig — (TIF) [file pntd.0003664.s004.tif]
